# STAT6 mutations enriched at diffuse large B-cell lymphoma relapse reshape the tumor microenvironment

**DOI:** 10.1007/s12185-023-03692-x

**Published:** 2024-01-29

**Authors:** Alexandre Benoit, Madelyn J. Abraham, Sheena Li, John Kim, Roger Estrada-Tejedor, Rowa Bakadlag, Nivetha Subramaniam, Kiran Makhani, Cynthia Guilbert, Raymond Tu, Matthew Salaciak, Kathleen Oros Klein, Krysta Mila Coyle, Laura K. Hilton, Raoul Santiago, Svetlana Dmitrienko, Sarit Assouline, Ryan D. Morin, Sonia V. del Rincon, Nathalie A. Johnson, Koren K. Mann

**Affiliations:** 1 Lady Davis Institute, Jewish General Hospital, 3755 Côte Sainte-Catherine Road, Montreal, QC H3T 1E2 Canada; 2 Division of Experimental Medicine, McGill University, Montreal, QC Canada; 3 University of British Columbia, Vancouver, BC Canada; 4 Organic and Pharmaceutical Chemistry Department, IQS School of Engineering, Universitat Ramon Llull, Barcelona, Spain; 5 Department of Pharmacology and Therapeutics, McGill University, Montreal, QC Canada; 6 Department of Molecular Biology and Biochemistry, Simon Fraser University, Burnaby, BC Canada; 7 Centre for Lymphoid Cancer, British Columbia Cancer, Vancouver, BC Canada; 8 Department of Pediatrics, Faculty of Medicine, Universite Laval, Quebec City, QC Canada; 9 Division of Pathology, McGill University Health Centre, Montreal, QC Canada; 10 Department of Oncology, McGill University, Montreal, QC Canada

**Keywords:** DLBCL, STAT6, Tumor microenvironment

## Abstract

Diffuse large B-cell lymphoma (DLBCL) relapses in approximately 40% of patients following frontline therapy. We reported that *STAT6*^*D419*^ mutations are enriched in relapsed/refractory DLBCL (rrDLBCL) samples, suggesting that JAK/STAT signaling plays a role in therapeutic resistance. We hypothesized that *STAT6*^*D419*^ mutations can improve DLBCL cell survival by reprogramming the microenvironment to sustain STAT6 activation. Thus, we investigated the role of STAT6^D419^ mutations on DLBCL cell growth and its microenvironment. We found that phospho-STAT6^D419N^ was retained in the nucleus longer than phospho-STAT6^WT^ following IL-4 stimulation, and STAT6^D419N^ recognized a more restricted DNA-consensus sequence than STAT6^WT.^ Upon IL-4 induction, *STAT6*^*D419N*^ expression led to a higher magnitude of gene expression changes, but in a more selective list of gene targets compared with STAT^WT^. The most significantly expressed genes induced by STAT6^D419N^ were those implicated in survival, proliferation, migration, and chemotaxis, in particular CCL17. This chemokine, also known as TARC, attracts helper T-cells to the tumor microenvironment, especially in Hodgkin’s lymphoma. To this end, in DLBCL, phospho-STAT6^+^ rrDLBCL cells had a greater proportion of infiltrating CD4^+^ T-cells than phospho-STAT6^−^ tumors. Our findings suggest that STAT6^D419^ mutations in DLBCL lead to cell autonomous changes, enhanced signaling, and altered composition of the tumor microenvironment.

**Supplementary Information:**

The online version contains supplementary material available at 10.1007/s12185-023-03692-x.

## Introduction

Diffuse large B-cell lymphoma (DLBCL) is the most common non-Hodgkin’s Lymphoma (NHL), accounting for 30–40% of all worldwide NHL cases [1]. DLBCL is a heterogenous disease, with several gene expression and mutational signatures. Germinal center B-cell (GCB) and activated B-cell (ABC) are the two major molecular subtypes of DLBCL, arising from different steps in B-cell activation and leading to distinct genomic signatures and clinical outcomes [2, 3]. Recently, DLBCL subcategories were re-defined by adding genetic aberrations to gene expression [4–6]; however, all DLBCL patients currently receive the same chemo-immunotherapy, R-CHOP: rituximab, cyclophosphamide, doxorubicin, vincristine, and prednisone [7]. Although R-CHOP shows significant efficacy, DLBCL relapses in approximately 40% of patients, and rrDLBCL is fatal in 90% of patients [8].

Mechanisms of DLBCL progression following R-CHOP treatment in vivo are unclear, but we hypothesized that mutations enriched in R-CHOP-resistant patients versus newly diagnosed patients play a central role in relapse [9]. Thus, we compared mutations found in R-CHOP-resistant patients with newly diagnosed patients to identify genes with a greater prevalence of mutation at relapse. One such recurrent mutation was in signal transducer and activator of transcription 6 (STAT6) at residue D419, found in 36% of relapsed/refractory GCB-DLBCL (rrDLBCL) and transformed lymphoma (rrTLy) samples [9]. STAT6 is a transcription factor frequently hyperactivated in follicular lymphoma [10], primary mediastinal large B-cell lymphoma (PMBCL) [11] and classical Hodgkin’s lymphoma (cHL) [12]. However, STAT6 hyperactivation in de novo DLBCL is rare, where *STAT6* mutation occurs in less than 5% of cases [9]. Despite identification of *STAT6* mutations in other lymphomas [10, 13, 14], little is known about how mutation changes its function.

The canonical STAT6 signaling pathway begins with IL-4 or IL-13 binding to their receptors, allowing for receptor autophosphorylation. This phosphorylation creates docking sites for the SH2 domain of STAT6, and recruits JAK proteins that phosphorylate STAT6 at Tyr641. Upon phosphorylation, STAT6 homodimerizes and translocates to the nucleus, where it binds DNA and acts as a transcriptional activator or repressor (reviewed by Goenka and Kaplan [15]). Upon activation, STAT6 has central roles in immune cell functions in normal and pathological conditions [16–19].

Here, we interrogated steps of the STAT6 signaling cascade in the context of the clinically-relevant D419 mutation to determine how mutated STAT6 function is altered to promote DLBCL cell survival. We asked whether (1) STAT6^D419^ mutants are constitutively active or have altered kinetics in response to cytokine stimulation, (2) STAT6^D419^ mutants have altered gene expression program or DNA binding, and (3) any changes in the tumor cell phenotype correlate with D419 mutation. In combination, our data reveals that STAT6^D419N^ has increased nuclear localization upon IL-4 stimulation, where binds to a restricted consensus sequence and promotes increased expression of target genes. Furthermore, STAT6^D419^ mutant tumor cells secrete more of the chemokine CCL17 upon IL-4 stimulation, and we are the first to provide evidence that this leads to increased CD4^+^ T-cell infiltration in rrDLBCL patient tumor biopsies.

## Materials and methods

### Cell culture and transfection

All cell line identities were confirmed by short tandem repeat analyses (Genetica Cell Line Testing, North Carolina). OCI-Ly8, SU-DHL-4, and DB GCB-DLBCL cells were cultured in Roswell Park Memorial Institute (RPMI) media containing 10% fetal bovine serum (FBS) and 0.5% penicillin–streptomycin (P/S). HEK293ft cells were cultured in Dulbecco’s Modified Eagle Medium (DMEM) supplemented with 10% FBS and 0.5% P/S. Transient transfection of HEK293ft cells was performed with Lipofectamine2000, as per manufacturer protocol (see Supplementary reagents and tools table for details of the material used in this work).

### Cloning and site-directed mutagenesis

pLX304-eGFP, pLX304-*STAT6* (NM_001178079.1), p3xFlag-CMV-14, pCMV-3-*IL-4* (NM_000589.3), and LeGO-iT plasmids were purchased. Using site-directed mutagenesis, four *STAT6*^*D419*^ mutated constructs were generated (D419A/G/H/N) in the pLX304 backbone, with a C-terminal V5 tag on each to distinguish between endogenous and overexpressed STAT6 upon transfection or transduction. *STAT6*^*D419N*^ was additionally chosen to be cloned into p3xFlag-CMV-14 for immunoprecipitation experiments by amplifying *STAT6* with NotI and XbaI restriction sites added, then digesting with NotI and XbaI and ligating into the same restriction sites in the p3xFlag-CMV-14 plasmid.

The pCMV-3-*IL-4* plasmid was used to amplify *IL-4,* with BamHI and NotI restriction sites added, and following digestion with BamHI and NotI restriction enzymes, the *IL-4* insert was ligated into LeGO-iT for generation of a stable IL-4 secreting stromal cell line.

Accuracy of cloning was confirmed via sanger sequencing at Genome Quebec.

### Generation of STAT6 mutant B-cell lymphoma cell lines

To generate stable STAT6 mutant human GCB-DLBCL cell lines, OCI-Ly8, SU-DHL-4, and DB cells were transduced with pLX304 lentiviruses expressing *STAT6*^*WT*^*, STAT6*^*D419A/G/H/N*^, or empty vector control.

### Cellular fractionation and western blotting

Cells were lysed in RIPA buffer to generate whole cell extracts (WCE). To fractionate cells, the cytoplasm was lysed in “Lysis Buffer B” (10 mM Tris at pH 8.4, 140 mM NaCl, 1.5 mM MgCl_2_, 0.5% IGEPAL, 1 mM DTT), and nuclear protein was further extracted using a detergent stock (3.3% sodium deoxycholate, 6.6% Tween40), RIPA buffer, and harsh sonication.

Protein was separated using 8% SDS-PAGE and transferred to nitrocellulose membranes. Membranes were blocked with 5% milk in TBS for 1 h and were incubated with primary antibody overnight at 4℃, followed by secondary antibody for 2 h at room temperature. Membranes were incubated for 1 min with ECL and exposed to film or imaged with a Bio-Rad ChemiDoc. Relative protein expression was calculated via densitometry, using ImageJ.

### Chronic IL-4 transwell co-culture model

HEK293ft cells were transduced with LeGO-iT or LeGO-iT-*IL-4* lentiviruses, and the resulting IL-4-secreting cells and empty vector control cells were named “293-IL-4” and “293-EV”, respectively. 293-IL-4 cells simulated chronic IL-4 induction using a transwell co-culture model, by seeding 293-EV or 293-IL-4 cells in a 6-well plate at day 1 and seeding OCI-Ly8-pLX304 cells overtop a 0.4 μm pore membrane at day 2. To model acute IL-4 stimulation, OCI-Ly8 cells in co-culture with 293-EV cells were stimulated with 200 pg/mL IL-4 for 3 h.

### Immunoprecipitation

Following transient transfection of HEK293ft cells with *STAT6*^*WT or D419N*^*-Flag* and *STAT6*^*WT or D419N*^*-V5,* nuclear protein was extracted with Nuclear Complex Co-IP Kit (Active Motif). Protein extracts (250 µg per condition) were pre-cleared using protein G agarose beads. For Flag pull-down, lysates were incubated overnight at 4 °C with M2 beads (Sigma), and for V5 pull-down, lysates were incubated overnight at 4 °C with 2 μg V5 antibody, then were incubated for 2 h at room temperature with magnetic dynabeads. For both pulldowns, beads were washed 3 times with lysis buffer following immunoprecipitation, then were boiled in 2 × Laemmli buffer prior to western blotting.

### RNA sequencing and ingenuity pathway analysis

RNA was extracted using the Absolutely Total RNA Purification Kit (Agilent). RNA sequencing was performed at Génome Québec (see supplemental methods for RNA sequencing analysis and Ingenuity Pathway Analysis).

### qPCR

RNA extracted using the E.Z.N.A. Total RNA Kit I (Omega Bio-Tek) was used for RT-PCR. cDNA was diluted 1/10 for qPCR with GoTaq qPCR master mix (Promega), using 100 nM primers. Transcript levels were normalized to three reference genes (*TBP*, *RPLP0*, and *HPRT1*), and fold change was calculated relative to empty vector control using the ΔΔCt method.

### Molecular modelling

Using the STAT6 DNA complex structure (PDBID: 4Y5W), loop modelling techniques were applied to predict the starting conformation of undefined loops (247–252, 324–336, 395–399) and were energy minimized. The system was prepared for molecular dynamics (MD) simulations by protonating all residues and correcting charge and hybridization states when needed. D419 residues were manually modified in both STAT6 monomer sequences using UCSF Chimera and new side chains were energy minimized.

Molecular dynamics simulations were conducted using AMBER20 software. Tleap module was used for neutralizing and solvating the system, defining a 10 Å truncated octahedron TIP3P water box. Solvent was energy minimized and heated to 300 K (200 ps, NTP) keeping the rest of the system restrained (defining 10 kcal/mol force constant). The whole system was therefore energy minimized to reduce the intermolecular clashes. Once the energy was minimized, non-water molecules were fixed again by applying positional restraints (10 kcal/mol) and the system was heated to 300 K (100 ps, NVT). A 100 ps NPT density equilibration stage, keeping the restraints, was performed before a progressive reduction of the restraints with four NVT equilibration stages (10, 8, 5, 1 kcal/mol). A final 100 ps NPT density equilibration without restraints was conducted before a 30 ns NPT production stage.

For umbrella sampling simulations, 50 independent molecular simulations (5 ns/window) were performed using AMBER20 software defining a movement of 1 Å in each window (force constant, *k* = 4 kcal/mol/Å). The Potential of Mean Force was calculated using WHAM (defining 1·10–5 tolerance).

### Binding motif analysis

STAT6 DNA-binding sequences were identified with the Gene Transcription Regulation Database (GTRD) [20]. The Analysis of Motif Enrichment (AME) [21] tool of the MEME Suite 5.4.1 was used to identify the presence of the STAT6 canonical consensus motif and the Multiple Em for Motif Enrichment (MEME) [22] tool was used to identify novel consensus motifs (see supplementary materials and methods).

### Enzyme-linked immunosorbent assay (ELISA)

293-EV or 293-IL-4 cells were seeded in a 6-well plate at day 1, and OCI-Ly8, DB, or SU-DHL-4 cells were seeded overtop a 0.4 μm pore membrane at day 2. On day 5, media was collected and snap frozen. CCL17 was quantified in the conditioned media using R&D human CCL17 DuoSet ELISA. Optical density (OD) values were calculated by subtracting the OD 540 nm from the OD 450 nm.

### CCL17-reporter assay

293-EV or 293-IL-4 cells were transfected with the CCL17-reporter plasmid containing the STAT6 responsive element upstream of the Firefly luciferase (fLuc) gene [23] (a kind gift from Dr. Daniel Hebenstreit, University of Warwick), a Renilla luciferase (rLuc) control plasmid (Promega), and either pLX304-EV, pLX304-STAT6^WT^, or pLX304-STAT6^D419N^. Twenty-four hours later, fLuc and rLuc activities were measured with a Dual-Luciferase assay kit (Promega). Relative luminescence units (RLU) were calculated by normalizing fLuc to rLuc.

### Patient data

The cohort used for microarray analysis and immunohistochemistry was previously described [9], and consisted of samples obtained prospectively from patients treated in Montreal QC and enrolled in the QCROC-2 clinical trial NCT01238692 or obtained from the Leukaemia Cell Bank of Quebec-Lymphoma axis. Patients provided informed consent for the clinical trial or for storage of samples in the databank. This project was approved by the research ethics board at the Jewish General Hospital, in accordance with the declaration of Helsinki (biobanking approval number 11-046 and STAT6 project 15-036).

An additional validation cohort was obtained from BC Cancer and consisted of matched transcriptomic and genomic data from de novo DLBCL patients [24–26] (see supplementary materials and methods for details).

### Immunohistochemistry

Immunohistochemistry (IHC) was performed using a Discovery XT Autostainer, with primary antibody incubation for 30 min at 37 °C. Tissues were counterstained with Hematoxylin, blued with Bluing Reagent, dehydrated through graded alcohols, cleared in xylene, and mounted. Sections were scanned at 20× using an Aperio AT Turbo Scanner. Staining intensity was scored by a blinded pathologist.

### Statistical analysis

Data was analyzed by unpaired Student’s *t* test, 1way ANOVA, or 2way ANOVA with Tukey’s post-hoc analysis. Sample size and statistical test are indicated in figure legends, where applicable.

### Data availability statement

Raw and processed RNA sequencing data are available at GEO accession number GSE220762.

## Results

### ***STAT6***^***D419***^ mutation neither increases the proliferative rate of GCB-type DLBCL cells nor STAT6 phosphorylation in response to IL-4

**Fig. 1:**
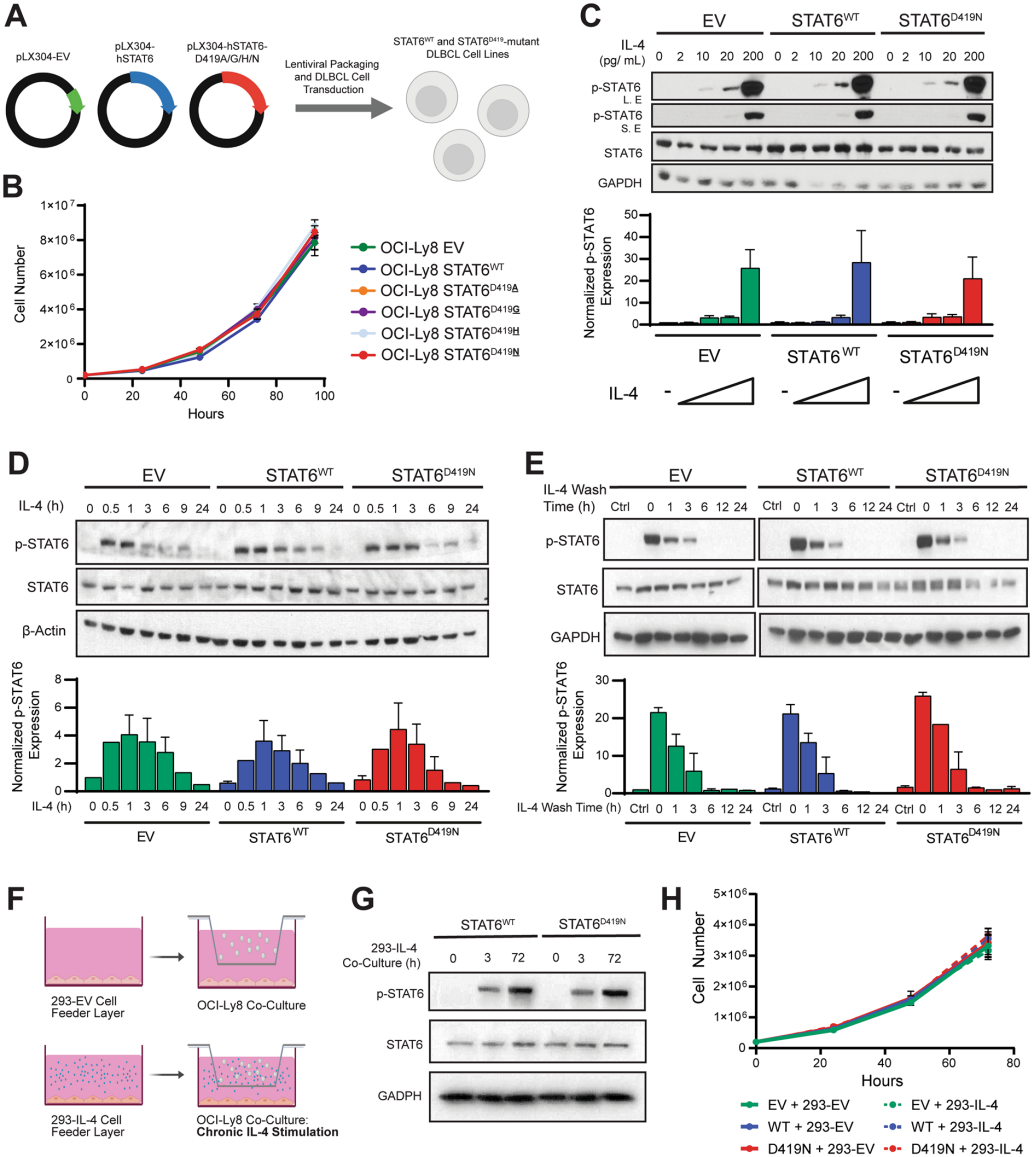
*STAT6*^*D419*^ cells have similar response to IL-4 as S*TAT6*^*WT*^ cells. **A** Schematic demonstrating the generation of STAT6 mutant DLBCL cell lines. **B** Growth curve of OCI-Ly8-pLX304 cell lines, expressing empty vector, STAT6^WT^, or STAT6^D419A/G/H/N^. Cells were counted once every 24 h for 96 h using a haemocytometer and trypan blue exclusion. Data shown are 4 biological replicates, consisting of 3 technical replicates each. **C** Representative western blot of 3 biological replicates demonstrating OCI-Ly8 sensitivity to increasing concentrations of IL-4 for 30 min. (S.E: Short exposure; L.E: Long exposure). **D** Representative western blot of 3 biological replicates demonstrating OCI-Ly8 time course of 200 pg/mL IL-4 stimulation. **E** Representative western blot of 3 biological replicates demonstrating OCI-Ly8 200 pg/mL IL-4 wash off time course. **F** Schematic illustrating the OCI-Ly8 and 293-EV/IL-4 co-culture system. **G** Western blot demonstrating that co-culture with 293-IL-4 induces sustained STAT6 phosphorylation. **H** Growth curve of OCI-Ly8 cell lines co-cultured with 293-EV or 293-IL-4 for 72 h. Data shown are 4 biological replicates, consisting of 2 technical replicates each. In all cases, bar graphs show densitometry of phospho-STAT6 expression, normalized to GAPDH or β-actin loading control expression

The enrichment of *STAT6*^*D419*^ mutations at relapse suggests they contribute to cell survival in DLBCL. Indeed, knock-down of *STAT6* in DLBCL, PMBCL, cHL, and FL reduces cell viability in the absence of IL-4, which suggests that STAT6 has tonic survival functions [13, 27, 28]. To test this, we generated OCI-Ly8 [29], SU-DHL-4 [30], and DB [31] cell lines mimicking the heterozygous expression of mutations found in patient biopsies by overexpressing *STAT6*^*WT*^ or *STAT6*^*D419*^ mutants (Fig. 1A). Each of these cell lines are known to be derived from GCB-type DLBCL, and each of their mutational profiles further suggests that they are of the EZB subtype, as defined by Wright et al. [4] and characterized by mutations in genes such as *EZH2, CREBBP,* and *KMT2D.* STAT6 mutations are typically associated with the EZB subtype, and our patient data further asserts that STAT6 mutant rrDLBCL is of the EZB subtype (Fig. S1). Overexpression of *STAT6*^*WT*^ or *STAT6*^*D419A/G/H/N*^ had no effect on DLBCL growth, as compared to empty vector controls (Figs. 1B, S2A). In addition, expression of *STAT6*^*D419*^ mutation alone had no impact on cellular response to any of the individual components of R-CHOP therapy (Fig. S2C). Together, these results indicate that *STAT6*^*D419*^ mutant cells do not have enhanced proliferation or increased therapeutic resistance in the absence of cytokine stimulation.

We observed enriched phospho-STAT6 staining in DLBCL patient biopsies with a *STAT6*^*D419*^ mutation [9], suggesting that these mutations lead to a constitutively active STAT6, altered kinetics of response to cytokine, or increased cytokine signaling from the microenvironment. To test the first two of these possibilities, phospho-STAT6 was assessed with increasing concentrations of IL-4. STAT6 was not phosphorylated in the absence of IL-4 (Fig. 1C), and each cell line had similar STAT6 phosphorylation in response to different concentrations of IL-4 (Fig. 1C). We next assessed whether STAT6^D419N^ mutation changed the kinetics of STAT6 phosphorylation. IL-4-induced phospho-STAT6 expression peaked between 30 min and 1 h, and gradually declined to background levels by 24 h in all cell lines (Figs. 1D, S2B). Furthermore, when cells were treated with IL-4 for 3 h and media was removed, the loss of phospho-STAT6 was similar between cell lines (Fig. 1E). These findings indicate that STAT6^D419N^ is not constitutively active nor is STAT6^D419N^ phosphorylation altered in response to IL-4. Of note, we found that exogenous expression of *STAT6*^*WT*^ and *STAT6*^*D419N*^ had no impact on total STAT6 expression levels in OCI-Ly8 and SU-DHL-4 cells (Figs. 1C, S2), suggesting that there is a buffering mechanism that keeps total STAT6 protein levels static.

Our data demonstrate that cytokine stimulation is required for mutant STAT6 phosphorylation. To test whether D419 mutation alters cell growth in the presence of sustained cytokine signaling, we developed a co-culture model wherein DLBCL cells are cultured in a transwell above a stromal cell line that constitutively produces IL-4 (293-IL-4; Fig. 1F), leading to sustained STAT6 phosphorylation (Fig. 1G). Using this co-culture system, OCI-Ly8, DB, and SU-DHL-4 expressing *STAT6*^*WT*^ or *STAT6*^*D419A/G/H/N*^ cells have a similar proliferative rate with and without IL-4 induction (Figs. 1H, S2D-F). Thus, IL-4 does not enhance the proliferative rate of *STAT6*^*D419*^ cells in vitro*.*

### STAT^D419N^ can heterodimerize with STAT6^WT^.

**Fig. 2:**
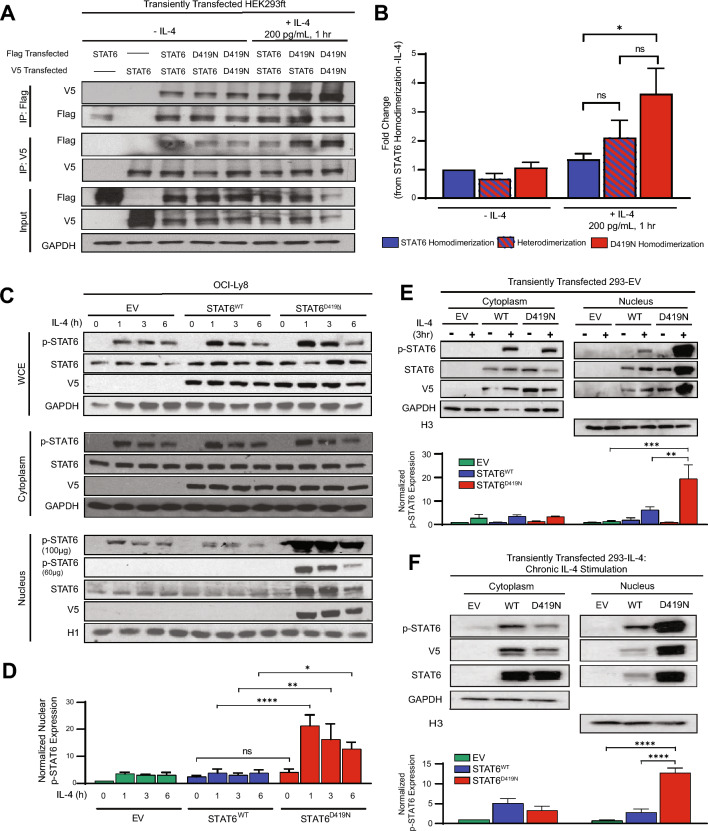
Increased presence of STAT6^D419N^ hetero- and homodimers in the nucleus upon IL-4 stimulation. **A** Representative western blot of 3 biological replicates from co-IP experiments. Lysates used for co-IP were from nuclear fractions. Labelling indicates transfection conditions. “Input” immunoblots are from whole cell extracts, to confirm equal transfection conditions. **B** Densitometry of Flag IP. Band intensity was normalized to input control, and then represented as fold change in intensity from the STAT6^WT^ homodimerization -IL-4 condition. **C** Representative western blot of 3 biological replicates from OCI-Ly8-EV, OCI-Ly8-STAT6^WT^, and OCI-Ly8-STAT6^D419N^ cellular fractions stimulated with 200 pg/mL IL-4 over 6 h. The nuclear fraction shows phospho-STAT6 nuclear accumulation with D419N mutation. **D** Densitometry of nuclear phospho-STAT6 expression, normalized to H1 loading control expression and expressed as fold change from the EV without IL-4. **E**, **F** Representative western blots of 3 biological replicates from cytoplasmic and nuclear cell fractions of transfected 293-EV cells stimulated with IL-4 for 3 h (acute stimulation; **E**) and transfected 293-IL-4 cells (chronic stimulation; **F**). Labelling indicates transfection conditions. Bar graphs show densitometry of p-STAT6 expression, normalized to GAPDH or H3 loading control. Bars in the graph are presented corresponding to western blot loading. Statistics on all bar graphs are 2-way ANOVA with multiple comparison (*p < 0.05, ** = *p* < 0.01, *** = *p* < 0.005, **** = *p* < 0.001)

In the STAT6 canonical signaling cascade, STAT6 dimerizes upon phosphorylation, and translocates to the nucleus [15]. This dimerization step is critical within the STAT6 activation cascade; thus, we questioned whether D419N mutation impacts the ability of STAT6 to form dimers and whether STAT6^D419N^ could dimerize with STAT6^WT^. To test the ability of STAT6^WT^ and STAT6^D419N^ to homo- and heterodimerize, we used co-immunoprecipitation (co-IP) in HEK293ft cells transiently expressing tagged *STAT6*^*WT*^ or *STAT6*^*D419N*^ capable of increasing phospho-STAT6 in response to IL-4 (Fig. S3). We transfected Flag-tagged and V5-tagged *STAT6*^*WT*^ or *STAT6*^*D419N*^ and stimulated cells for 1 h with IL-4. By immunoprecipitating with anti-Flag and probing for V5, and the reciprocal, we found that STAT6^WT^ and STAT6^D419N^ can both homodimerize and heterodimerize (Fig. 2A). Interestingly, STAT6^WT^-STAT6^D419N^ heterodimers and STAT6^D419N^ homodimers were immunoprecipitated from nuclear extracts more readily upon IL-4 stimulation, as compared to STAT6^WT^ homodimers (Fig. 2B). The latter suggests an increased nuclear localization of STAT6 dimers when one or both monomers contain the D419N mutation.

### STAT6^D419N^ displays increased nuclear translocation.

Next, we assessed STAT6^D419N^ phosphorylation kinetics in cellular fractions following IL-4 stimulation in OCI-Ly8 cell lines. Cells were stimulated with IL-4 for 1, 3, and 6 h, and phospho-STAT6 expression was assessed in whole cell, cytoplasmic, and nuclear protein extracts. In whole cell extracts, phospho-STAT6 induction by IL-4 was similar between STAT6^WT^ and STAT6^D419N^, consistent with our initial findings (Figs. 1D, 2C). Likewise, within the cytoplasmic fraction, phospho-STAT6 was similar between *STAT6*^*WT*^ and *STAT6*^*D419*^ mutant cells. However, within the nuclear fraction, STAT6^D419N^ displayed increased phosphorylation in response to IL-4 (Fig. 2C–D). Similar results were obtained with SU-DHL-4 cells (Fig. S4A) and when *STAT6*^*D419N*^ mutant OCI-Ly8 cells were stimulated with IL-13 (Fig. S4B). We also confirmed HEK293ft cells transiently transfected with *STAT6*^*D419N*^ have increased nuclear expression both upon acute (3 h; Fig. 2E) and chronic (72 h; Fig. 2F) exposure to IL-4. Thus, STAT6^D419^ mutants have increased nuclear expression following cytokine activation, concordant with patient data showing strong nuclear phospho-STAT6 staining [9].

### STAT6^D419N^ upregulates a more stringent set of genes with increased magnitude

**Fig. 3:**
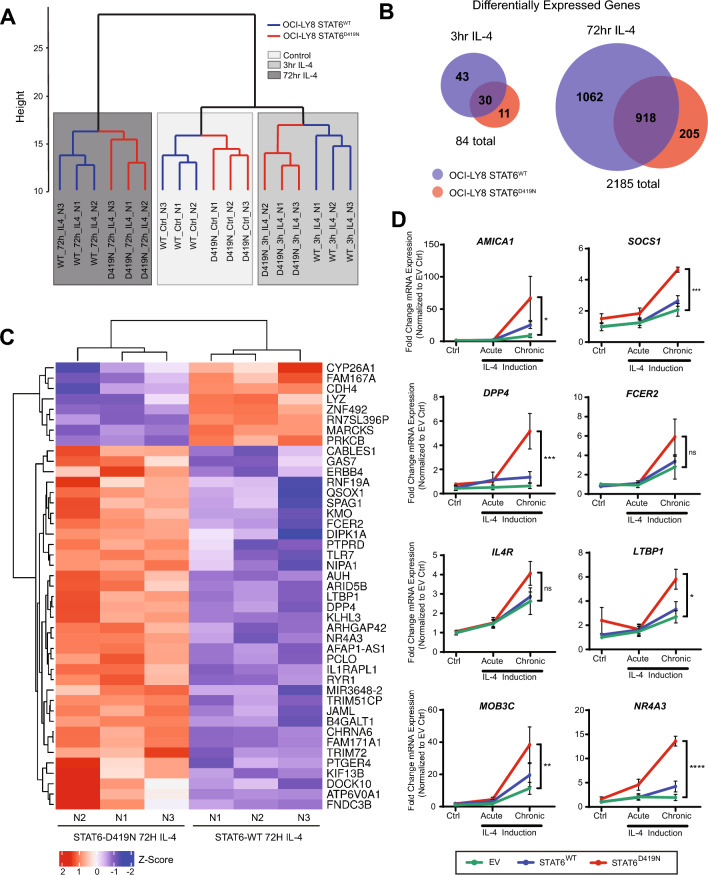
STAT6^D419N^ upregulates a restricted set of genes with increased magnitude. **A** Dendrogram demonstrating that OCI-Ly8-STAT6^WT^ and OCI-Ly8-STAT6^D419N^ groups cluster by IL-4 treatment time. **B** Venn diagram showing the number of DEGs upon 3 h and 72 h IL-4 treatment. Upon IL-4 treatment, STAT6^WT^ has more unique DEGs than STAT6^D419N^. **C** Heatmap showing gene expression differences between STAT6^WT^ and STAT6^D419N^ cells, upon 72 h IL-4 treatment. Blue or red intensity represents Z-score. **D** qPCR validations of RNAseq data demonstrate that STAT6^D419N^ upregulates transcription of gene targets with increased magnitude. Data consist of 4 biological replicates, with 3 technical replicates each (2way ANOVA; *p < 0.05, ** = *p* < 0.01, *** = *p* < 0.005, **** = *p* < 0.001)

We next hypothesized that the increased nuclear expression of STAT6^D419N^ might lead to altered transcription factor activity and thereby, altered gene expression. To assess differential gene expression signatures, we extracted RNA from OCI-Ly8 cells expressing either *STAT6*^*WT*^ or *STAT6*^*D419N*^ grown in co-culture with 293-EV or 293-IL-4 cells for 3 h (acute stimulation) or 72 h (chronic stimulation) and performed RNA sequencing. In the absence of IL-4, gene expression between *STAT6*^*WT*^ and *STAT6*^*D419N*^ was similar and clustered by exposure time rather than genotype by hierarchical clustering (Fig. 3A). Only 4 genes were differentially expressed in the absence of IL-4 between *STAT6*^*WT*^ and *STAT6*^*D419N*^ cells, including *PRKCB, IL1RAPL1, ZNF492,* and *RN7SL396P*. We compared the IL-4-dependent differentially expressed genes (DEGs) between *STAT6*^*WT*^ and *STAT6*^*D419N*^ cells and identified only 84 DEGs after acute IL-4 treatment and 2,185 DEGs after 72 h (Fig. 3B). Of note, the DEGs which distinguished STAT6^WT^ and STAT6^D419N^ cells following 72 h IL-4 treatment were often upregulated in STAT6^D419N^ as compared to STAT6^WT^ (Fig. 3C). However, we also found that IL-4 induced DEGs are often shared by both cell lines (35% in acute and 42% in chronic exposure; Fig. 3B), and include the STAT6 transcriptional targets *FCER2, IL4R, AMICA1, SOCS1,* and *DPP4*. Using qPCR, we validated the expression of these genes, as well as some novel genes found to be upregulated by IL-4 induction, such as *NR4A3, LTBP1, and MOB3C*. Each of these genes were upregulated in *STAT6*^*WT*^ and *STAT6*^*D419N*^ cells upon chronic IL-4 stimulation, and gene expression of *SOCS1, DPP4,* and *NR4A3* was induced more in STAT6^D419N^ cells than in STAT6^WT^ cells (Fig. 3D). Furthermore, expression of each of these genes depends on sustained IL-4 signaling, as transcript levels decline when IL-4 is removed from cell culture media, leading to pre-stimulation levels by 6–12 h post-IL-4 wash off (Fig. S5).

Interestingly, there were more DEGs exclusive to *STAT6*^*WT*^ than *STAT6*^*D419N*^ cells at both time points. After chronic IL-4 exposure, there were 5 × more DEGs selective for *STAT*^*WT*^ (1062 in WT vs 205 in D419N; Fig. 3B). These results were surprising, given that STAT6^D419N^ demonstrated enhanced nuclear localization. Together, our RNA sequencing and qPCR data demonstrate that STAT6^D419N^ upregulates the expression of a more selective gene set, but with increased magnitude, upon IL-4 stimulation.

### STAT6^D419^ mutation does not uniformly impact DNA-binding dynamics.

**Fig. 4:**
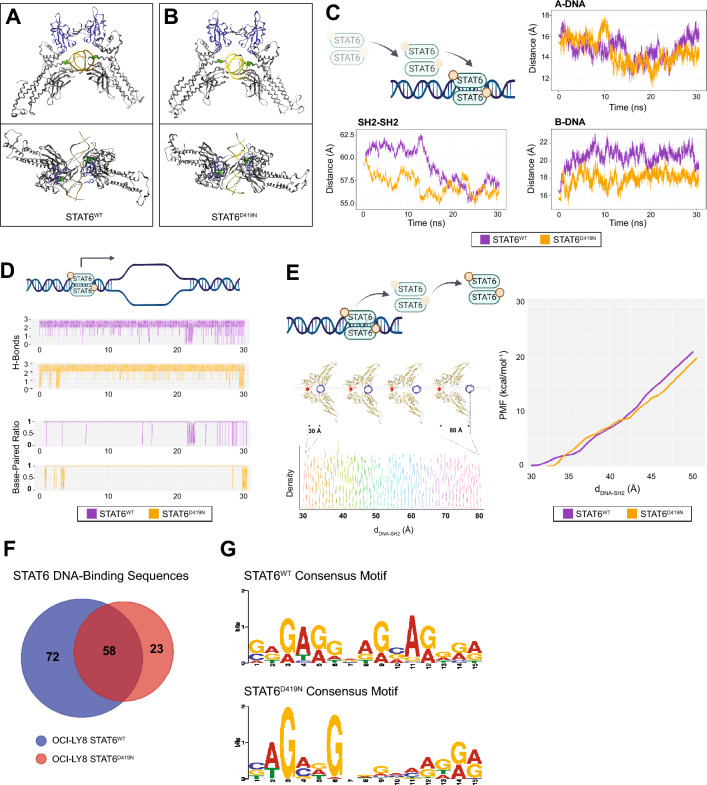
STAT6^D419N^ recognizes an altered DNA-consensus motif. **A**, **B** Ribbon diagrams showing the DNA-bound conformation of STAT6^WT^ and STAT6^D419N^. **C** Molecular dynamic simulations showing STAT6 DNA-binding dynamics. SH2-SH2 shows the distance between SH2 domains measured between their centre of mass, A-DNA shows the distance between one of the D419 residues and the DNA centre of mass (chain A, resID 291), and B-DNA shows the distance between the other D419 residue and the DNA centre of mass (chain B, resID 814), during the process of STAT6^WT^ or STAT6^D419N^ binding to DNA. **D** Evolution of the number of DNA intermolecular hydrogen bonds and the paired bases ratio along the MD simulation. When purple and orange lines jump from one limit to the other, it denotes a higher ratio of frames without H-bonds and a lower BP-ratio. **E** Graphical representation of the umbrella sampling study, and Potential of Mean Force (PMF) obtained after umbrella sampling simulations. The distance between SH2-SH2 and DNA was considered as the collective variable under study. Fifty independent simulations were conducted for each STAT6 DNA complex to explore all the molecular states when changing the collective variable from 30 to 80 Å. The overlap in density plots along the coordinate assure the thermodynamic reliability of the study. PMF approximates the free energy landscape when following a coordinate of interest (a.k.a. collective variable). On the right, the PMF involving the DNA unbinding process is shown. **F** Venn diagram showing the number of identified STAT6-binding sequences identified within proximity of genes upregulated by STAT6^WT^ or STAT6^D419N^ upon 3 h IL-4 treatment. **G** STAT6^WT^ and STAT6^D419N^ novel consensus motifs, as identified with MEME using STAT6 DNA-binding sequences obtained from GTRD

The D419 residue locates along the major groove of the protein-DNA-binding interface, and it has therefore been previously predicted that mutation of the D419 residue may influence DNA binding by STAT6 [10, 13]. Thus, we next questioned how D419 mutation of *STAT6* might alter DNA interactions. When MD simulations were used to model the molecular structure of endogenous DNA-bound STAT6^WT^ (Figs. 4A, S6A) and STAT6^D419N^ (Fig. 4B), no remarkable differences in the backbone geometry were observed. Likewise, MD were also used to describe STAT6 DNA binding by monitoring the evolution of three distances: first, the distance between both SH2 domains; second, the distance between D419 residue and DNA centres of mass (chain A); and third, the distance with DNA chain B. No major differences were found for STAT6^WT^ and STAT6^D419N^ systems (Figs. 4C, S6B, C). Once bound, STAT6^WT^ and STAT6^D419N^ showed similar ability to unravel the DNA structure, as illustrated by the predicted number of hydrogen bonds between base pairs and the base paired ratio (Figs. 4D, S6D). Furthermore, umbrella sampling simulations were used to model STAT6 release from the DNA. Starting from the stable DNA-bound conformation, the DNA structure was pulled out of the STAT6 structure, by increasing the distance from SH2-SH2 and DNA from 30 to 80 Å, until the DNA was released from the protein. These simulations found that STAT6 released from DNA around 50 Å, and D419N mutation did not induce a change in the free energy of binding (Figs. 4E, S6E). While STAT6^D419N^ showed no remarkable differences in DNA-binding dynamics from STAT6^WT^, STAT6^D419A^ and STAT6^D419G^ did have significantly changed DNA binding affinity (Fig. S6), with STAT6^D419A^ and STAT6^D419G^ showing greater ability to unravel the DNA secondary structure, and STAT6^D419G^ additionally showing an increase in the free energy required for DNA unbinding. These findings indicate that D419 mutation does not uniformly change STAT6 DNA interactions, and that increased DNA-binding affinity does not necessarily underlie the increased nuclear presence of STAT6 mutants or the increased transcription of STAT6 target genes.

### STAT6^D419N^ recognizes a restricted DNA-consensus sequence.

STAT6^D419N^ regulates the expression of a more stringent set of genes than STAT6^WT^, so we next questioned whether there was a difference in DNA-binding motifs recognized by STAT6^WT^ and STAT6^D419N^. Genes upregulated upon 3 h of IL-4 stimulation are likely to be primary targets of STAT6 transcription factor activity, thus, this dataset was used for this analysis. ChIP-seq data compiled by GTRD [20] were used to identify STAT6 DNA-binding sequences upstream and downstream of genes upregulated in STAT6^WT^ only (72 sequences), STAT6^D419N^ only (23 sequences), and in both STAT6^WT^ and STAT6^D419N^ (58 sequences); thus 130 STAT6-binding sequences were associated with STAT6^WT^, and 81 STAT6-binding sequences were associated with STAT6^D419N^ (Fig. 4F). Within these STAT6-binding sequences, the canonical palindromic STAT6 5′-TTC(N)_3_…_4_GAA-3′ consensus motif [32] was identified in 10% of STAT6^WT^-associated sequences (13/130; *E*value = 9.29e−1), and in 8.6% of STAT6^WT^-associated sequences (7/81; *E*value = 6.14e−1), serving as an internal validation of this methodology, but also suggesting the canonical STAT6-binding consensus motif is not significantly enriched within our dataset. However, a novel DNA-consensus sequence motif was identified within our dataset (Fig. 4G) at 70 sites with an *E* value of 1.5e−014 in STAT6^WT^-associated binding sequences and at 59 sites with an *E* value of 5.9e−005 in STAT6^D419N^-associated binding sequences. Interestingly, this binding motif is restricted to a guanine residue at positions 3 and 6 in sequences regulated by STAT6^D419N^ (G-NN-G motif), while the residues at positions 3 and 6 are more variable in sequences regulated by STAT6^WT^. These results suggest that the binding of STAT6^D419N^ to DNA is restricted to a more specific sequence than STAT6^WT^.

### STAT6^D419^ mutants upregulates CCL17 expression to recruit CD4 + T-Cells

**Fig. 5:**
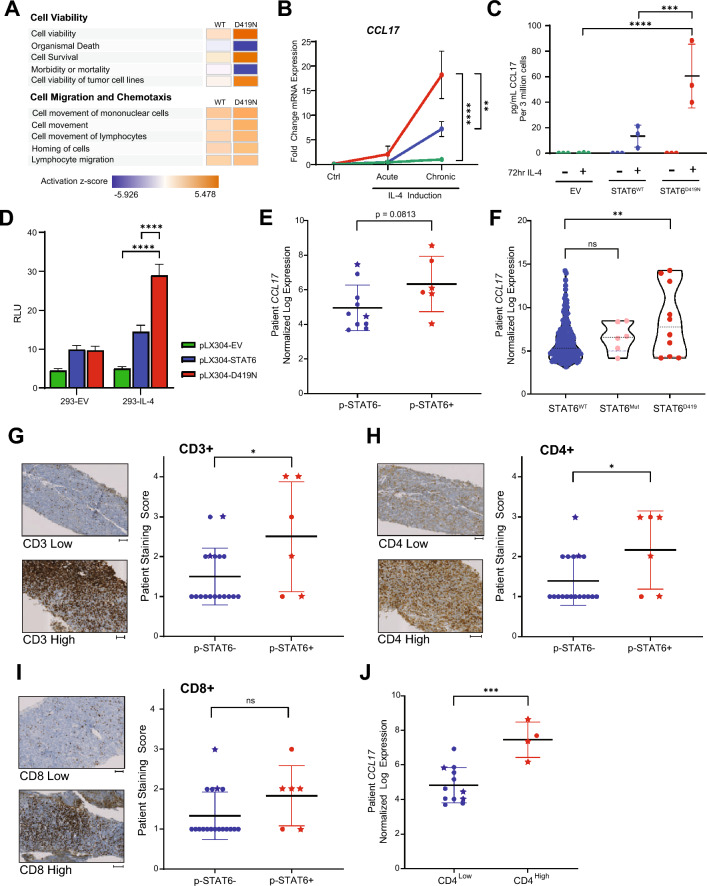
*STAT6*^*D419*^ mutation results in increased CCL17 secretion and increased invasion of CD4 T-Cells. **A** IPA analysis of RNAseq data showing STAT6^D419N^ cells have enrichment in pathways related to Cell Viability and Cell Migration and Chemotaxis. **B** qPCR showing *CCL17* transcription is increased in STAT6^D419N^ cells upon IL-4 stimulation. Data consist of 4 biological replicates in technical triplicate (2-way ANOVA; ****p < 0.001). **C** ELISA showing CCL17 secretion is increased in STAT6^D419N^ cells compared to STAT6^WT^ cells upon IL-4 stimulation. Data consists of 3 biological replicates in technical triplicate (2-way ANOVA; **p < 0.01). **D** CCL17-reporter luciferase assay showing CCL17 transcription is increased 30-fold when STAT6^D419N^ is expressed in 293-IL-4 cells. (RLU: relative luminescence units). **E*** CCL17* expression obtained from gene microarray in GCB rrDLBCL patients who had negative or positive phospho-STAT6 staining (two-tailed unpaired *t* test; *p < 0.05). Stars indicate the presence of a STAT6^D419^ mutation. **F** *CCL17* expression obtained from a compendium of RNA sequencing of 598 de novo DLBCL patients and 1 transformed lymphoma patient, comparing *CCL17* expression in GCB STAT6^WT^ patients with *CCL17* expression in STAT6 non-D419 mutant patients (STAT6^Mut^) and STAT6^D419^ mutant patients (1-way ANOVA; **p < 0.01). Of the STAT6^Mut^ patients, 4 were GCB and 2 were ABC. Of the STAT6^D419^ mutant patients, 9 were GCB and 1 was unclassified **G**–**I** CD3, CD4, and CD8 staining in GCB rrDLBCL patient biopsies. CD3, CD4, or CD8 staining intensity score was compared between phospho-STAT6 negative and phospho-STAT6 positive patients (scored by a blinded pathologist; 1 = negative staining, 2 = weak staining, 3 = moderate staining, 4 = strong staining). Scale bars below each image indicate 100 µm. Stars indicate the presence of a STAT6^D419^ mutation. **J** *CCL17* expression obtained from gene microarray in GCB rrDLBCL patients who had low CD4 staining (score 1–2), or high CD4 staining (score 3–4; unpaired *t* test; ***p < 0.005). Stars indicate the presence of a STAT6^D419^ mutation

To further understand the DEGs selective to STAT6^WT^ or STAT6^D419N^, we performed pathway analyses of the genes significantly altered in *STAT6*^*WT*^ or *STAT6*^*D419N*^ OCI-Ly8 cells upon chronic IL-4 induction. We found that *STAT6*^*D419N*^ cells have increased activation of pathways that have genes related to cell viability and survival, and less activation of pathways that have genes related to organismal death and cell morbidity/mortality as compared to *STAT6*^*WT*^, suggesting that *STAT6*^*D419N*^ cells have improved survival in response to IL-4. Notably, we found that expression of *STAT6*^*D419A/G/H/N*^ does not confer a proliferative advantage in GCB-DBCL cell lines, although these cell lines are culture-adapted and highly proliferative, potentially masking a mutation-induced phenotype. *STAT6*^*D419N*^ cells also have increased activation in pathways related to chemotaxis and cell migration, including cell movement and migration of mononuclear cells and lymphocytes (Fig. 5A; Table 1). Together, these data indicate that *STAT6*^*D419N*^ cells have a survival advantage in response to IL-4 stimulation and may have increased capability to attract or migrate to specific immune cells that could be IL-4 secreting.

**Table 1:** IPA analysis of OCI-Ly8-STAT6^WT^ and OCI-Ly8-STAT6^D419N^ cells under chronic IL-4 stimulation

Pathway	Number of genes in pathway	STAT6^WT^		STAT6^D419N^	
		*Z-score*	Overlap *p* value	*Z-score*	Overlap *p* value
Cell viability					
Cell viability	311	1.475	1.63E−26	5.478	5.10E−11
Organismal death	445	− 0.633	5.98E−19	− 0.593	8.36E−08
Cell survival	328	1.03	9.47E−28	5.232	1.04E−10
Morbidity or mortality	451	− 0.307	2.85E−19	− 5.506	2.56E−10
Cell viability of tumor cell lines	208	0.695	8.28E−20	4.544	5.94E−08
Cell migration and chemotaxis					
Cell movement of mononuclear cells	102	2.403	3.81E−10	3.421	1.04E−15
Cell movement	436	1.683	6.38E−22	3.259	3.04E−26
Cell movement of lymphocytes	89	1.951	5.31E−10	2.954	1.98E−15
Homing of cells	119	1.906	4.27E−10	2.795	2.45E−13
Lymphocyte migration	78	1.98	3.95E−09	2.698	9.50E−14

*STAT6*^*D419*^ mutant rrDLBCL patient biopsies have increased phospho-STAT6 staining and an increased STAT6 expression signature [9], suggesting that *STAT6*^*D419*^-mutants are able to recruit IL-4 secreting cells to maintain an activated state. This aligns with the pathway analyses showing enrichment in chemotactic signals. One interesting candidate from our dataset and a known STAT6 transcriptional target is *CCL17* (aka TARC), a chemokine that can attract various CD4^+^ T-cells, such as Th2 cells and Tregs. CCL17 is upregulated in human FL samples that express mutated STAT6 [10, 13], but this has not been shown in STAT6 mutant DLBCL cell lines. To this end, qPCR determined that IL-4-induced *CCL17* mRNA expression is significantly upregulated by STAT6^D419A/G/H/N^ as compared to control, in OCI-Ly8, SU-DHL-4, and DB cells (Figs. 5B, S7A-C). In addition, CCL17 was more highly secreted by *STAT6*^*D419A/G/H/N*^ than *STAT6*^*WT*^ cells following chronic IL-4 induction (Figs. 5C, S7D-F). Using a CCL17-reporter luciferase assay in 293-EV and 293-IL-4 cells, we confirmed the upregulation of CCL17 by STAT6^D419N^ was indeed transcriptional (Fig. 5D).

We assessed whether this observation could be translated to clinical samples, using our previously published gene microarray data [9] from CD19 + cells selected from rrDLBCL biopsies that were also annotated for phospho-STAT6 expression, indicating STAT6 activation by IL-4. Patients were stratified into “phospho-STAT6 positive” and “phospho-STAT6 negative” groups, with the “phospho-STAT6 positive” group enriched in *STAT6*^*D419*^ mutations, but also including mutations in other members of the STAT6 signaling axis (i.e., IL4R, SOCS1, and JAK2). This allowed us to expand our analyses to all rrDLBCL samples with the same phenotype. We found that phospho-STAT6 positive rrDLBCL tumors had increased *CCL17* mRNA expression (Fig. 5E). In addition, using an independent cohort from BC Cancer, where phospho-STAT6 histology data was not available, *CCL17* expression was compared in STAT6^WT^ and STAT6^D419^ mutant patient samples. Although these samples were obtained from de novo and not relapsed/refractory patients, CCL17 was still found to be significantly increased when a D419 mutation was present (Fig. 5F). Furthermore, in this dataset, CCL17 expression was not found to be increased in patients with a non-D419 STAT6 mutation, indicating a unique role for the mutated D419 residue.

Given the role of secreted CCL17 as a chemoattractant, we hypothesized that the surrounding microenvironment of phospho-STAT6 positive tumors might be enriched in T-cells. Using serial sections from rrDLBCL biopsies, we assessed the presence of CD3, CD4, and CD8 antigens with IHC, and found that phospho-STAT6 positive rrDLBCL tumors have a significantly higher proportion of CD3^+^ T-cells (Fig. 5G). Half of phospho-STAT6 positive tumors had a high percentage (25–50%) of CD4^+^ T-cells, and of the 3 phospho-STAT6^+^/CD4^High^ samples, 2 had a *STAT6*^*D419*^ mutation (Fig. 5H). Moreover, even the phospho-STAT6 negative samples with a *STAT6*^*D419*^ mutation were found to have high infiltration of CD4^+^ T-cells. Interestingly, there were no significant differences in the presence of CD8^+^ T-cells when samples were stratified by phospho-STAT6 status (Fig. 5I). However, when we stratified the data by STAT6 mutation, CD8^+^ T-cells were significantly increased in STAT6 mutant biopsies, in addition to the CD4^+^ T-cells (Fig. S8). Finally, to establish a link between CCL17 in rrDLBCL tumor cells and CD4^+^ T-cell infiltration, *CCL17* mRNA expression was compared between CD4^High^ and CD4^Low^ samples, and there was a striking increase in tumor cell *CCL17* mRNA expression in samples with CD4^High^ staining (Fig. 5J). Together, these results indicate that STAT6 mutant rrDLBCL cells remodel their tumor microenvironment (TME) via the secretion of the chemokine CCL17 to attract CD4^+^ T-cells, although the subtype of CD4^+^ T-cells which were recruited were not determined in this study.

## Discussion

STAT6 mutations are enriched at relapse in DLBCL and localize to the D419 hotspot within the DNA-binding domain [9], implying that mutations in the *STAT6*^*D419*^ DNA-binding domain are functionally significant in rrDLBCL. Given that relapse in DLBCL is considerable, and the mechanisms behind tumor growth post-R-CHOP are poorly elucidated, understanding how *STAT6*^*D419*^ mutations contribute to DLBCL relapse is an important clinical matter. Thus, our study used exogenously expressed *STAT6*^*WT*^ and *STAT6*^*D419A/G/H/N*^ in 3 different GCB-DLBCL cell lines to examine how STAT6 signaling is altered by D419 mutation. Upon IL-4 stimulation, STAT6^D419N^ has increased nuclear localization, perhaps recognizing a restricted DNA-consensus sequence, and resulting in a more stringent gene expression profile. Although molecular modeling did not indicate altered DNA binding, we only modeled the canonical STAT6 consensus sequence and did not account for altered nuclear protein expression. In addition to DNA binding, we hypothesize that increased nuclear localization may be due to 1) altered nuclear import or export [33]; or 2) altered protein–protein interactions contributing to nuclear retention.

Yildiz et al., previously observed that STAT6^D419G^ mutant cells exhibited nuclear STAT6 expression independent of IL-4 stimulation, but phosphorylation of STAT6 is still cytokine dependent [13]. Also, Yildiz et al. did not find global basal transcriptional differences compared to STAT6^WT^, which is consistent with our findings, suggesting that nuclear presence alone does not stimulate transactivation activity. Furthermore, Mentz et al. reported that STAT6^D419H^ mutations can be characterized as gain-of-function, but the mutant STAT6 protein still requires IL-4 stimulation for transactivation [10]. We have extended these data, showing that while mutant STAT6 hyperactivates some targets, STAT6^D419N^ has a more restricted set of target genes than the wild-type protein. STAT6 hyperactivation has been previously attributed to autocrine IL-13 production [34], JAK2 amplification [11], and a combination of autocrine IL-4/IL-13 production and overall enrichment of microenvironmental cytokines [12, 35–38]. In our hands, *STAT6*^*D419N*^ does not have increased nuclear residency in the absence of IL-4, nor do mutant GCB-DLBCL cells display evidence of autocrine IL-4/IL-13 induction or JAK2 co-mutation or amplification [9]. Thus, we predict that STAT6 activation in STAT6 mutant rrDLBCL is due to cytokine secretion from a constituent of the TME.

**Fig. 6:**
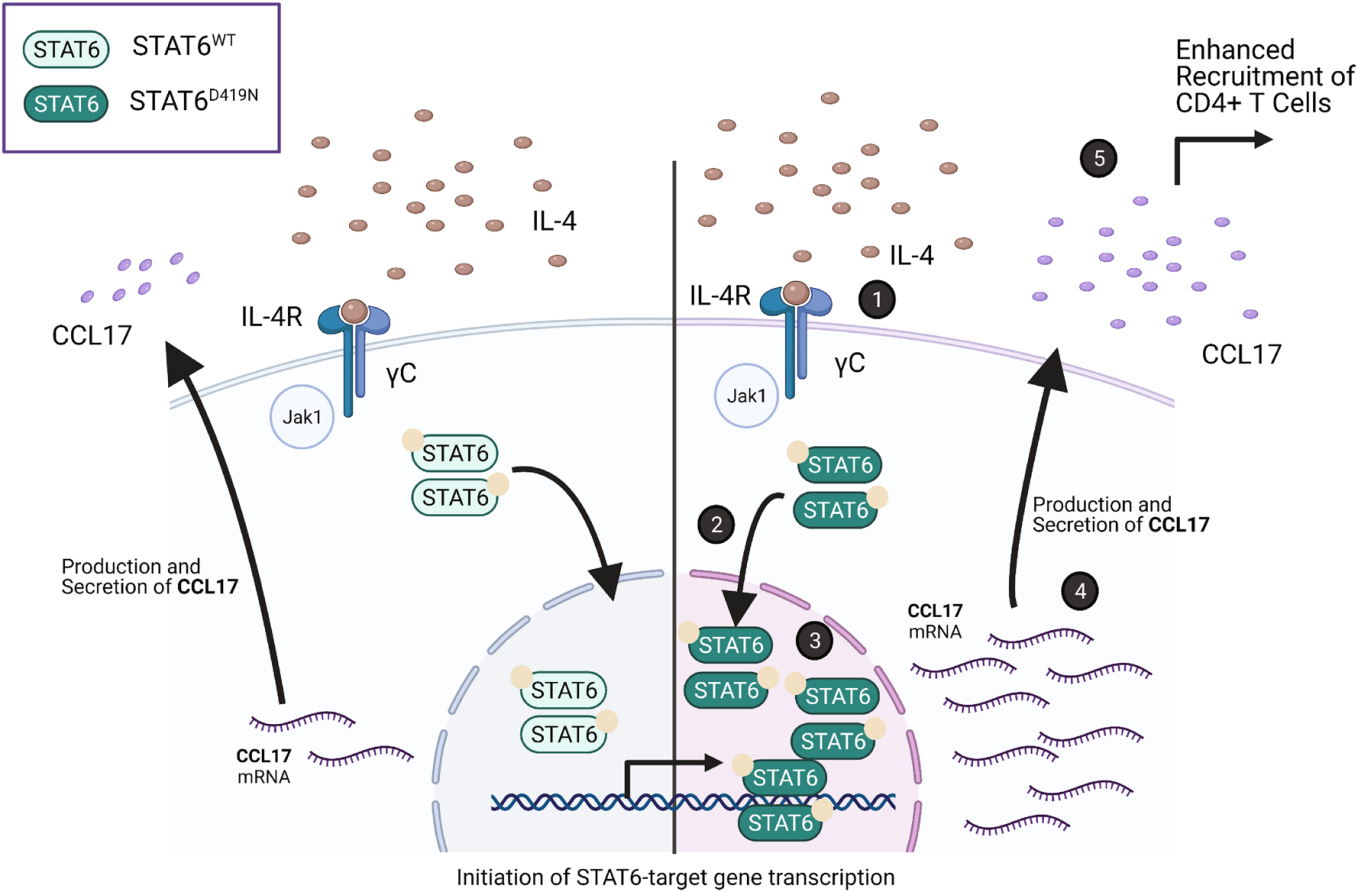
Schematic Summary of Data. **1**. In *STAT6*^*WT*^ and *STAT6*^*D419N*^ cells, IL-4 is required for STAT6 activation. **2.** Upon activation with IL-4, both STAT6^WT^ and STAT6^D419N^ will dimerize and translocate to the nucleus. **3.** However, STAT6^D419N^ has increased nuclear retention as compared to STAT6^WT^ and recognizes an altered DNA-consensus sequence. **4.** STAT6^D419N^ has increased transcription of select gene targets, such as *CCL17.* **5.** Increased transcription of *CCL17* by STAT6^D419N^ leads to increased production and secretion of CCL17, which functions to recruit CD4^+^ T-cells

STAT6^D419^ mutations have been previously investigated in the context of FL, which is a slow-growing lymphoma that relies on the TME for cancer cell survival [39]. While it has been shown that STAT6^D419^ mutation leads to upregulated CCL17 and CCL22 in FL patient samples [10], our group is the first to characterize STAT6^D419^ mutations in the context of rrDLBCL. rrDLBCL is the most deadly DLBCL, and is often characterized by an immune “depleted” TME [40]. However, STAT6 mutations in DLBCL are associated with a GC-type TME that is enriched in various CD4 T-cells [40]. To this end, in our rrDLBCL patient samples, phospho-STAT6 positivity and *STAT6*^*D419*^ mutation correlates with increased *CCL17* expression. CCL17 is a known STAT6 transcriptional target [41] and chemoattractant that recruits various CCR4 + immune cells—such as CD8^+^ or CD4^+^ T-cells, and macrophages—in murine and canine cancer models, in addition to human tumors [42–45]. While CCL17 is not normally secreted by B cells, it has previously been reported that expression can be induced [46, 47]. Indeed, in our study, IL-4 stimulation of STAT6 mutant cells induced an upregulation of *CCL17* expression, and furthermore, in patient samples, increased *CCL17* expression correlated with increased invasion of CD4^+^ T-cells. Therefore, we postulate that *STAT6*^*D419*^-induced-expression of CCL17 may recruit CD4^+^ T-cells capable of producing IL-4/IL-13, including Th2, Tfh, and Treg subsets, to maintain STAT6 activation (Fig. 6: Schematic Summary). Furthermore, we predict that there may be additional cascading impacts on the TME. For instance, if the CD4 + T-cell infiltrate is indeed IL-4/IL-13 secreting, this may have functional consequences on the non-tumor cells of the TME.

We speculated that the STAT6 mutant protein plays a role in therapeutic resistance, however, we found that STAT6^D419A/G/H/N^ mutations in GCB-DLBCL cells have no impact on cellular response to the individual components of R-CHOP. Thus, STAT6 mutations don’t directly cause resistance, but there is ample evidence that the TME is prognostic in DLBCL [40, 48, 49]. To this end, further investigation is warranted to determine if therapeutic resistance is associated with STAT6^D419^-induced microenvironmental remodeling. From a therapeutic angle, knowing how the tumor influences the surrounding immune cells is important, as cellular therapies, such as CAR-T [50, 51], are being increasingly used to treat rrDLBCL. Future studies may examine if STAT6 mutations affect responses to these novel therapies, by using immunocompetent mouse models that allow for the careful dissection and manipulation of the TME. Overall, our data show that tumor cell autonomous changes in STAT6 result in microenvironmental changes, and we believe further investigation could have an impact on informing standard of care for rrDLBCL patients, and potentially other B-cell malignancies identified with STAT6 mutations [10, 13, 14], by identifying novel therapeutic susceptibilities.

### Supplementary Information

Below is the link to the electronic supplementary material.

Supplementary file1 (DOCX 4078 kb)

## Data Availability

All data is available upon request.
