# Supplementary material for: STAT6 mutations enriched at diffuse large B-cell lymphoma relapse reshape the tumor microenvironment
Source: Int J Hematol. 2024 Jan 29;119(3):275–90. doi: 10.1007/s12185-023-03692-x (PMC10920476; doi:10.1007/s12185-023-03692-x)
Supplement: Supplementary file 1 — Supplementary file1 (DOCX 4078 kb) [file 12185_2023_3692_MOESM1_ESM.docx]

**STAT6 Mutations Enriched at Diffuse Large B-Cell Lymphoma Relapse Reshape the Tumour Microenvironment**

Alexandre Benoit*, Madelyn J. Abraham*, Sheena Li, John Kim, Roger Estrada-Tejedor, Rowa Bakadlag, Nivetha Subramaniam, Kiran Makhani, Cynthia Guilbert, Raymond Tu, Matthew Salaciak, Kathleen Oros Klein, Krysta Mila Coyle, Laura K. Hilton, Raoul Santiago, Svetlana Dmitrienko, Sarit Assouline, Ryan D. Morin, Sonia V. del Rincon, Nathalie A. Johnson, and Koren K. Mann.

**SUPPLEMENTAL MATERIALS AND METHODS**

**Lentivirus production and infection**

HEK293ft packaging cells were transfected with psPAX2, pMD2.G, and pLX304 plasmids containing *STAT6^WT^*, *STAT6D419^A/G/H/N^*, or empty vector control, or LeGO-iT plasmids containing *IL-4* or empty vector control. The lentivirus supernatant was filtered (0.45 µm), snap frozen, and kept at -80℃. To transduce OCI-Ly8 and SUDHL-4 cells with pLX304 viruses, spinoculation at 800xg for 30 minutes at room temperature with 6μg/mL polybrene was performed every 24hr for two consecutive days. The resulting cell lines were selected with 6µg/mL blasticidin for 5 consecutive days. To transduce HEK293ft cells, LeGO-iT viruses were added to cells with 6μg/mL polybrene every 24hr for two consecutive days. Transduced cells were selected via tdTomato positivity and were sorted into a 96 well plate with a BD FACSAria Fusion Cell Sorter at the Lady Davis Institute Flow Cytometry Facility.

**MTT Assay**

OCI-Ly8 cells were plated at 2.5x10^4^ cells per well in a 96-well plate, and were treated with increasing concentrations of Rituximab, Maphosphamide, Doxorubicin, Vincristine, or Dexamethasone. After 48 hours, cells were resuspended in 50uL of 5 mg/mL MTT and were incubated at 37°C for 4 hours. Following incubation, 150µL of acidified isopropanol (4 mM HCl and 0.1% NP40 in isopropanol) was added to each well, and absorbance was read at 590nm within 1 hour.

**RNA Sequencing Analysis and Ingenuity Pathway Analysis**

RNAseq data from paired read 100 bp sequencing runs (performed on the Illumina NovaSeq 6000 S2 PE100) was processed using the STAR aligner to the HG38 build of the human genome. Duplicate reads were not removed. Raw counts were obtained using the HTSeq Software and were filtered for low read depth. Genes that had a total read count below 10 across all samples were removed. Raw counts were normalized using the variance stabilizing transformation (‘vst’) function in the bioconductor package ‘DESEQ2’. To remove batch effect, the ‘removeBatchEffect’ function from the bioconductor package ‘limma’ was used. Differential Expression analysis was performed using the Bioconductor package ‘DESeq2’. Gene expression data was scaled by row to Z-scores, and ComplexHeatmap (1) was used for gene expression visualization.

The differentially expressed genes between STAT6^WT^ and STAT6^D419N^ under control, acute, and chronic IL-4 induction were analyzed via Ingenuity Pathway Analysis (IPA) software (Qiagen). Gene expression in acute and chronic conditions were compared to their respective controls, filtered by a p-adjusted value of < 0.05. Filtered genes were analyzed in IPA using the downstream effect analysis “diseases and bio functions” to identify biological processes that were altered, ordered by the magnitude of z-score difference.

**Binding Motif Analysis**

From RNAseq data, genes which were upregulated by STAT6^WT^ and STAT6^D419N^ upon 3hr IL-4 stimulation were identified. With this gene set, the Gene Transcription Regulation Database (GTRD) was used to identify STAT6 DNA-binding sequences which were within 5kb of each upregulated gene. The number of STAT6 DNA-binding peaks for each gene ranged from 0 to 17, with sequence length ranging from 25 to 96bp long. Using the Analysis of Motif Enrichment (AME) tool of the MEME Suite 5.4.1, our identified STAT6 DNA-binding sequences were aligned to the HOCOMOCO Human (v11 Full) motif database, to identify the presence of the STAT6 canonical consensus motif. Fisher’s exact test was used to test for motif enrichment and the *E*-value threshold for reporting enriched motifs was set to 15. The Multiple Em for Motif Enrichment (MEME) tool of the MEME Suite 5.4.1 was used to identify novel consensus motifs within our dataset. The Classic motif discovery mode was used, with motif distribution set to zero or one motif per sequence, and sequence width set to 6 – 15bp. To determine significance, we used an *E*-value metric, which is an estimate of the number of motifs with the same widths and number of occurrences which would have an equal or higher log likelihood ratio of occurring if the input sequences had been randomly generated.

**Analysis of Matched Whole Genome and RNA Sequencing**

Analysis was performed on a compendium of whole genome sequencing of 146 tumors and whole exome sequencing of 463 tumors from previously published studies (2-6). This included 608 *de novo* DLBCL and 1 transformed FL (post-histologic transformation). Mutations were detected as previously described (7) Briefly, mutations were called using the SLMS-3 pipeline that amalgamates Strelka (8), SAGE, Lofreq (9), and mutect2 (10) to report somatic variants supported by at least three algorithms. The consistent cutoff to call the variants was >=4 reads, and >=0.1 VAF. The detected variants were further annotated using command line vcf2maf (version 1.6.18) and Variant Effect Predictor (cache version 86).

Matched RNA sequencing data was available from 598 of the 609 tumors. Gene expression levels were quantified from fastq with Salmon (11) and normalized with DESeq2 (12) and variance stabilizing transformation. Expression matrices underwent batch correction to remove artifacts related to the source material: cohort, frozen whole tissue, immunotubes, and FFPE.

For analysis, samples were binned based on COO (ie. GCB vs ABC). As STAT6^D419^ mutations are exclusively found in GCB DLBCL, STAT6^WT^ GCB DLBCL cases were used for comparison. 10 cases had STAT6 D419 mutations (9 GCB; 1 unclassified) while 6 cases had other STAT6 mutations (4 GCB; 2 ABC).

**SUPPLEMENTAL REAGENTS AND TOOLS TABLE**

| **Reagent or resource** | **Reference or source** | **Identifier** |
| --- | --- | --- |
| **Experimental models: Cell lines** | | |
| OCI-Ly8 | Dr. Riccardo Dalla-Favera | N/A |
| SU-DHL-4 | ATCC | CRL-2957 |
| DB | ATCC | CRL-2289 |
| HEK293ft | ATCC | CRL-1573 |
| 293-EV | This paper | N/A |
| 293-IL-4 | This paper | N/A |
| **Recombinant DNA** | | |
| pLX304-eGFP | GeneCopoeia | EX-eGFP-LX304 |
| pLX304-*STAT6*  (NM_001178079.1) | GeneCopoeia | EX-OL01611-LX304 |
| pLX304-*STAT6^D419A/G/H/N^* | This paper | N/A |
| psPAX2 | Addgene | 12260 |
| pMD2.G | Addgene | 12259 |
| pCMV-3-*IL-4*  (NM_000589.3) | Sino Biological | HG11846-UT |
| LeGO-iT | Addgene | 27361 |
| LeGO-iT-*IL-4* | This paper | N/A |
| p3xFlag-CMV-14 | Sigma | E7908 |
| p3xFlag-CMV-*STAT6* | This paper | N/A |
| p3xFlag-CMV-*STAT6^D419N^* | This paper | N/A |
| pTATA-LUC-CCL17-cdWT | Dr. Daniel Hebenstreit | N/A |
| pRL | Promega | E2241 |
| **Antibodies** | | |
| Phospho-Stat6 (Tyr641)  Rabbit polyclonal (1:1000 for W.B.) | Cell Signaling | 9361 |
| V5 Tag  Rabbit polyclonal (1:5000) | Bethyl Laboratories | A190-120A |
| Stat6 Rabbit polyclonal (1:1000) | Cell Signaling | 9362 |
| Flag M2  Mouse monoclonal (1:2000) | Sigma | F3165 |
| GAPDH  Rabbit monoclonal (1:1000) | Cell Signaling | 2118 |
| Histone H3  Rabbit polyclonal (1:1000) | Cell Signaling | 9715 |
| β-actin  Mouse monoclonal (1:10,000) | Sigma-Aldrich | A5441 |
| Rabbit IgG  HRP Linked Whole Ab (1:3000) | GE Healthcare | NA934 |
| Mouse IgG  HRP Linked Whole Ab (1:5000) | GE Healthcare | NA931 |
| CD3  Rabbit monoclonal | Ventana Medical Systems | 790-4341 |
| CD4  Rabbit monoclonal | Ventana Medical Systems | 790-4423 |
| CD8  Rabbit monoclonal | Ventana Medical Systems | 790-4460 |
| OmniMap anti-Rabbit-HRP | Ventana Medical Systems | 760-4311 |
| **Oligonucleotides and other sequence-based reagents** | | |
| **PCR primers** | **Sequence** | **Direction** |
| *IL-4*-BamHI site | AACGGATCCATGGGTCTCACCTCCCAA | Forward |
| *IL-4*-NotI site | CCTGTTGCGGCCGCTCAGCTCGAACACTTTGA | Reverse |
| *STAT6-*NotI site | TAAGCTTGCGGCCGCATGTCTCTGTGG | Forward |
| *STAT6-*XbaI site | GATCCTCTAGAGCACCAACTGGGGTTGGC | Reverse |
| **Site-directed mutagenesis** | **Sequence** | **Direction** |
| *STAT6^D419A^*-5' | GTCCATGGCAACCAAGCCAACAATGCCA | Forward |
| *STAT6^D419^*^A^-3' | GGCTTTGGCATTGTTGGCTTGGTTGCCAT | Reverse |
| *STAT6^D419G^*-5' | GTCCATGGCAACCAAGGCAACAATGCCA | Forward |
| *STAT6^D419G^*-3' | GGCTTTGGCATTGTTGCCTTGGTTGCCAT | Reverse |
| *STAT6^D419H^*-5' | ATCGTCCATGGCAACCAACACAACAATG | Forward |
| *STAT6^D419H^*-3' | CTTTGGCATTGTTGTGTTGGTTGCCATGG | Reverse |
| *STAT6^D419N^*-5' | CATCGTCCATGGCAACCAAAACAACAAT | Forward |
| *STAT6^D419N^*-3' | GCTTTGGCATTGTTGTTTTGGTTGCCATG | Reverse |
| **qPCR primers** | **Sequence** | **Direction** |
| *TBP*-5' | TGCCACGCCAGCTTCGGAGA | Forward |
| *TBP*-3' | ACCGCAGCAAACCGCTTGGG | Reverse |
| *RPLP0*-5' | TCCTCGTGGAAGTGACATCGT | Forward |
| *RPLP0*-3' | CTGTCTTCCCTGGGCATCA | Reverse |
| *CCL17*-5' | ACCAATGTGGGCCGGGAGTG | Forward |
| *CCL17*-3' | GGCCCTGCCCTGCACAGTTA | Reverse |
| *AMICA1*-5' | TTCAGTGCATTGCCTCCCCTGG | Forward |
| *AMICA1*-3' | ACATGGACTGTTAGCTCAGGCGG | Reverse |
| *FCER2*-5' | GGGCTGCTGACTCTGCTTCT | Forward |
| *FCER2*-3' | TCTTCCAGCTGTTTTAGACTCTGTGT | Reverse |
| *DPP4*-5' | GGCACCTGGGAAGTCATCGGG | Forward |
| *DPP4*-3' | CGGATTCAGCTCACAACTGAGGC | Reverse |
| *SOCS1*-5' | CCCCTGGTTGTTGTAGCAG | Forward |
| *SOCS1*-3' | GTAGGAGGTGCGAGTTCAGG | Reverse |
| *LTBP1*-5' | AGCTGTGAGAAGGGGAACACCAC | Forward |
| *LTBP1*-3' | TGGCACCATGGACTGGGATCTG | Reverse |
| *NR4A3*-5' | TCAGCGCGCAAGATACCCTCC | Forward |
| *NR4A3*-3' | CTGCGCCGCATAACTGGAACC | Reverse |
| *MOB3C*-5' | CCACGCGTGTTGGAGTTCCC | Forward |
| *MOB3C* -3' | GGTGTTGACGTGCGCCTCTG | Reverse |
| *IL-4R*-5' | CCGGACGGCGAATGGAGCA | Forward |
| *IL-4R*-3' | GCAAAGCCACCCCATTGGGAGAT | Reverse |
| **TaqMan** | **Source** | **Identifier** |
| Human HPRT1 (HGPRT) Endogenous Control (FAM/MGB probe, non-primer limited) | Applied Biosystems | 4333768F |
| **Chemicals, enzymes, recombinant proteins, and other reagents** | | |
| BamHI | Roche | 10567604001 |
| NotI | Thermo Fisher Scientific | ER0595 |
| XbaI | NEB | R0145M |
| T4 DNA Ligase | NEB | M0202 |
| DMEM | Multicell | 319-005-CL |
| RPMI | Multicell | 319-000-CL |
| Fetal bovine serum | Multicell | 080450 |
| Penicillin-streptomycin | Multicell | 450-201-EL |
| β-mercaptoethanol | Sigma | M3148-100mL |
| Polybrene | Millipore | TR-1003-G |
| Blasticidin | Wisent | 400-190-EM |
| Protease Inhibitor Cocktail | Roche | 04693116001 |
| PhosphoStop Inhibitor Cocktail | Roche | 04906837001 |
| ECL chemiluminescent detection reagent | GE Healthcare | RPN2106 |
| Recombinant human IL-4 | PeproTech | 200-04 |
| Recombinant human IL-13 | PeproTech | 200-13 |
| Protein G Agarose | Abcam | ab193258 |
| Anti-Flag M2 Affinity Gel | Sigma | A2220 |
| Dynabeads Protein G for Immunoprecipitation | Thermo Scientific | 10004D |
| TaqMan Fast Universal PCR Master Mix (2X), no AmpErase UNG | Applied Biosystems | 4352046 |
| ChromoMap-DAB | Ventana Medical System | 760-159 |
| **Critical commercial assays** | | |
| Nuclear Complex Co-IP Kit | Active Motif | 54001 |
| E.Z.N.A. Total RNA Kit I | Omega Bio-tek | R6834-02 |
| Absolutely Total RNA Purification Kit | Agilent Technologies | 400800 |
| iScript cDNA Synthesis Kit | Bio-Rad | 170-8891 |
| qPCR Kits: GoTaq qPCR and RT-qPCR Systems | Promega | A6001 |
| Human CCL17/TARC DuoSet ELISA | R&D Systems | DY364-05 |
| DuoSet ELISA Ancillary Reagent Kit 2 | R&D Systems | DY008 |
| Human TARC ELISA | Ray Biotech | Q92583 |
| Dual Luciferase Reporter Assay System | Promega | E1910 |
| **Software and algorithms** |  |  |
| MEME Suite 5.4.1 | NIH |  |
| AMBER20 |  |  |
| Prism (version 6.0) | GraphPad |  |
| Illustrator (CS6) | Adobe |  |
| ImageJ (version 2) | NIH  https://imagej.net/Welcome |  |
| Aperio Image Scope (version 12.3.3) | Leica Bio system  https://www.leicabiosystems.com/digital-pathology/manage/aperio-imagescope/ |  |
| **Other** |  |  |
| BD FACSAria Fusion Cell Sorter | BD Bioscience |  |
| BD LSRFortessa Flow Cytometer | BD Bioscience |  |
| Falcon 6-well TC-treated Polystyrene Permeable Support Companion Plate, with Lid, Sterile | Corning | 353502 |
| Falcon Permeable Support for 6-well Plate with 0.4 µm Transparent PET Membrane, Sterile | Corning | 353090 |
| NanoDrop Spectrophotometer ND-1000 | NanoDrop Technologies |  |
| NovaSeq6000 S2 PE100 | Illumina |  |
| Eppendorf Mastercycler pro PCR System | Eppendorf |  |
| 7500 Fast Real-Time PCR System | Applied-Biosystems |  |
| EnSpire Multimode Plate Reader | PerkinElmer |  |
| Discovery XT Autostainer | Ventana Medical System |  |
| Aperio AT Turbo Scanner | Leica Biosystems |  |
| Bio-Plex 200 Systems | Bio-Rad |  |

**SUPPLEMENTAL FIGURES**

**
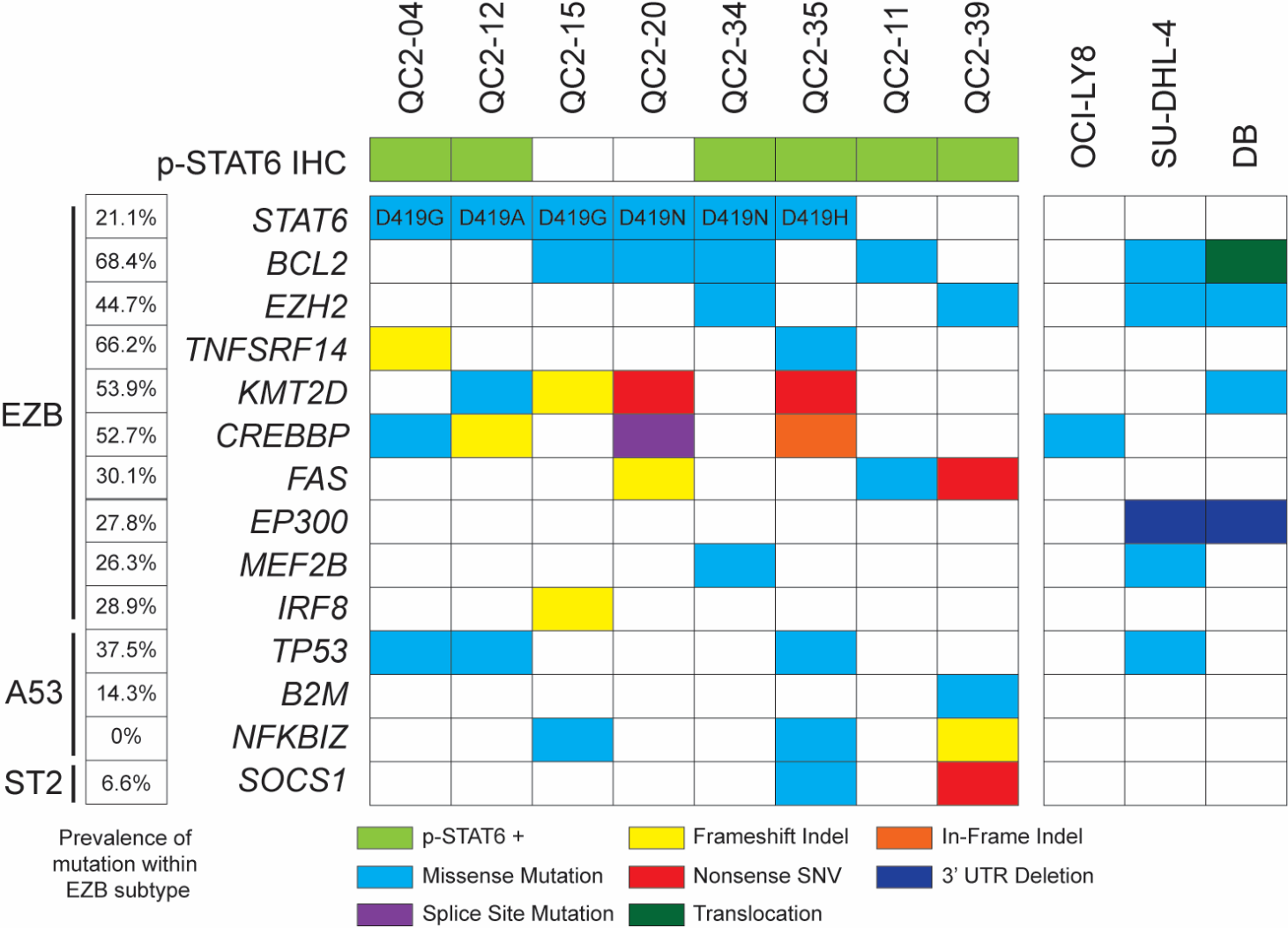
**

**Figure S1:**

Graph showing the mutational status of select genes in STAT6^D419^ mutant DLBCL, p-STAT6+ DLBLC, and the GCB-DLBCL cell lines used in this study. Mutational status of patients has been previously reported (13). In this cohort, patients with a STAT6^D419^ mutation and patients with p-STAT6 positivity by IHC always show co-mutation with genes that are characteristic of the EZB subtype of DLBCL (14). Accordingly, cell lines with similar mutational profiles were selected for use in this study.


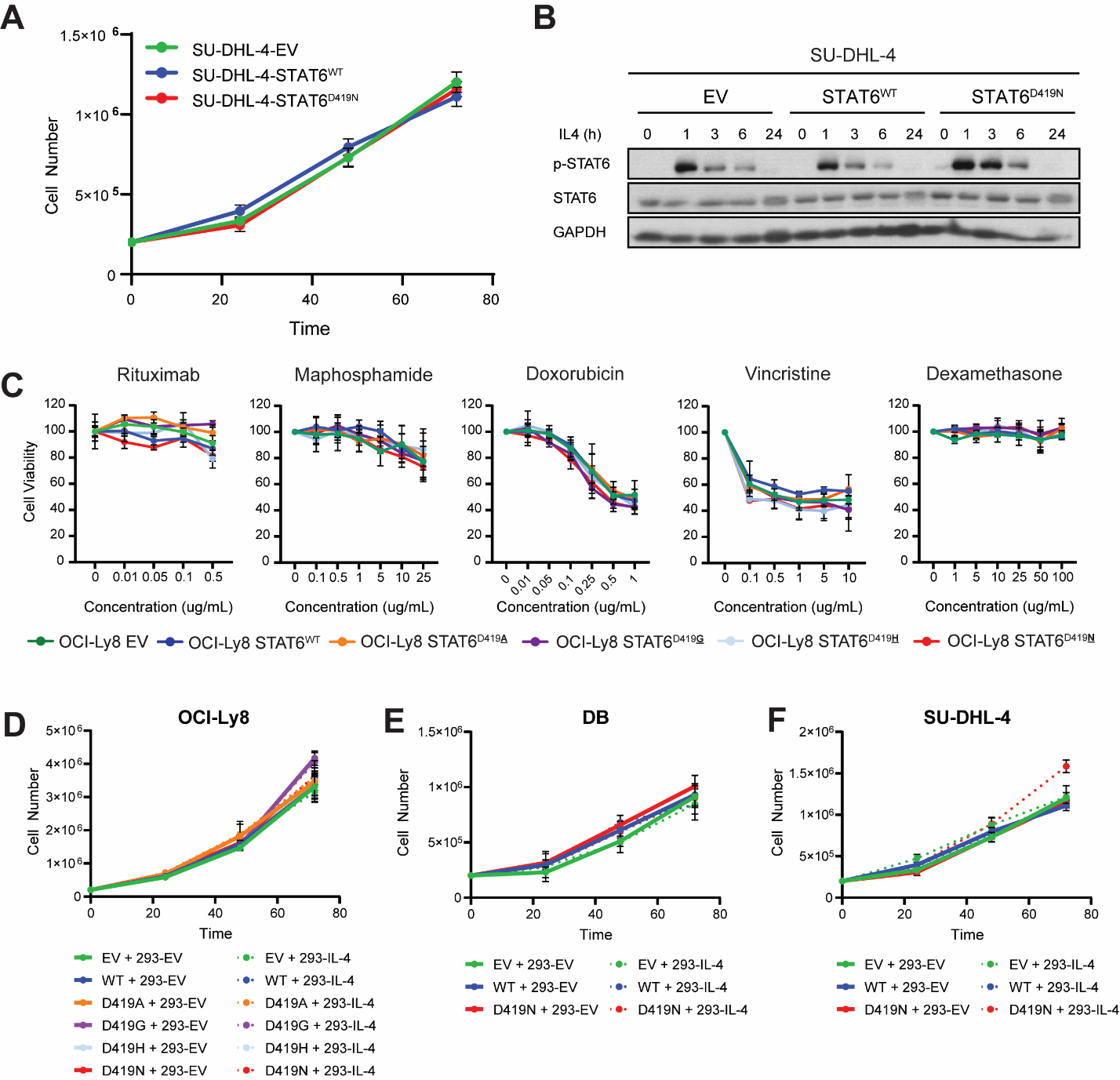


**Figure S2**

**A.** Growth curve of SU-DHL-4-pLX304 cell lines, expressing empty vector, *STAT6^WT^*, or *STAT6^D419N^*. Cells were counted once every 24hr for 72hr, using a haemocytometer and trypan blue exclusion. Data shown is 4 biological replicates. **B.** Representative western blot demonstrating SU-DHL-4 time course of IL-4 stimulation. **C.** Viability of OCI-Ly8 cells treated for 48hr with increasing concentrations of Rituximab, Maphosphamide, Doxorubicin, Vincristine, and Predisone. Data is for each drug is normalized to viability of untreated control. Data shown is 3 biological replicates consisting of 4 technical replicates each. **D-F.** Growth curves of OCI-Ly8 (**D**), DB (**E**), and SU-DHL-4 (**F**) cell lines co-cultured with 293-EV or 293-IL-4 for 72 hours. Data shown are 3 biological replicates, consisting of 2 technical replicates each.


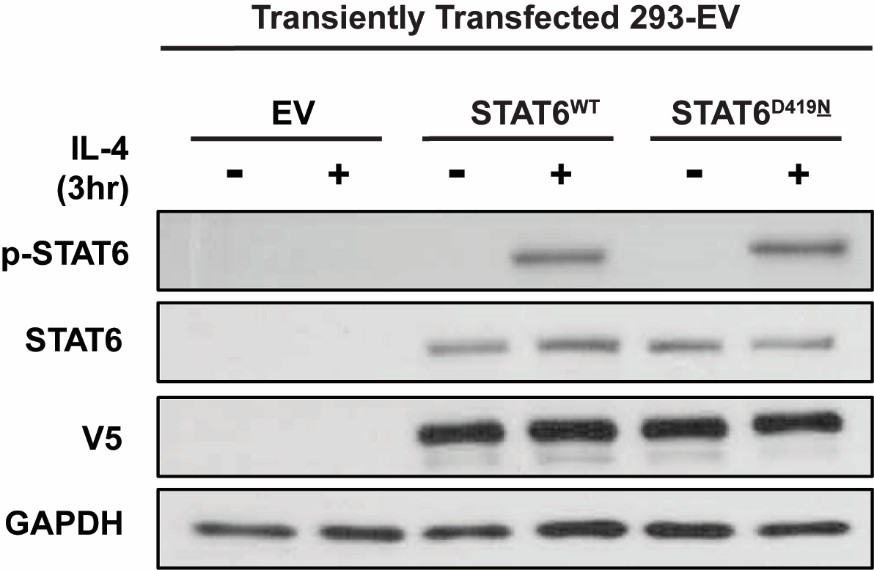


**Figure S3:**

Representative western blot from 3 biological replicates confirming that 293-EV cells transfected with pLX304-*STAT6^WT^* or pLX304-*STAT6^D419N^* are responsive to IL-4 stimulation, as indicated by phospho-STAT6 induction.


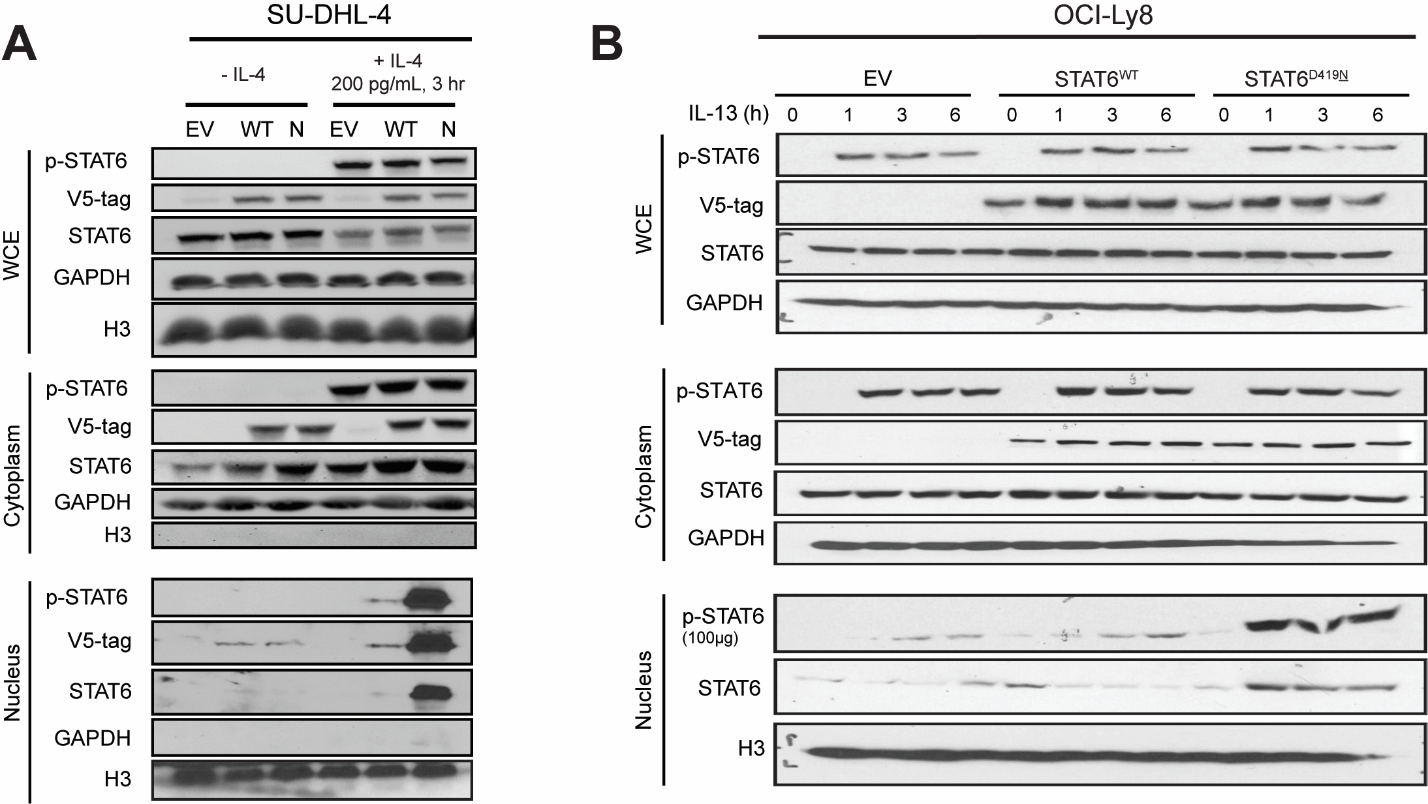


**Figure S4:**

**A.** Representative western blot from 3 biological replicates demonstrating increased nuclear expression of STAT6 in SU-DHL-4-*STAT6^D419N^* cells upon 3 hours of IL-4 stimulation. **B.** Representative western blot from 3 biological replicates from OCI-Ly8-*STAT6^D419N^* showing that IL-13 stimulation of OCI-Ly8-*STAT6^D419N^* cells has a similar phenotype as IL-4 stimulation: increased nuclear accumulation as compared to *STAT6^WT^* and empty-vector control.


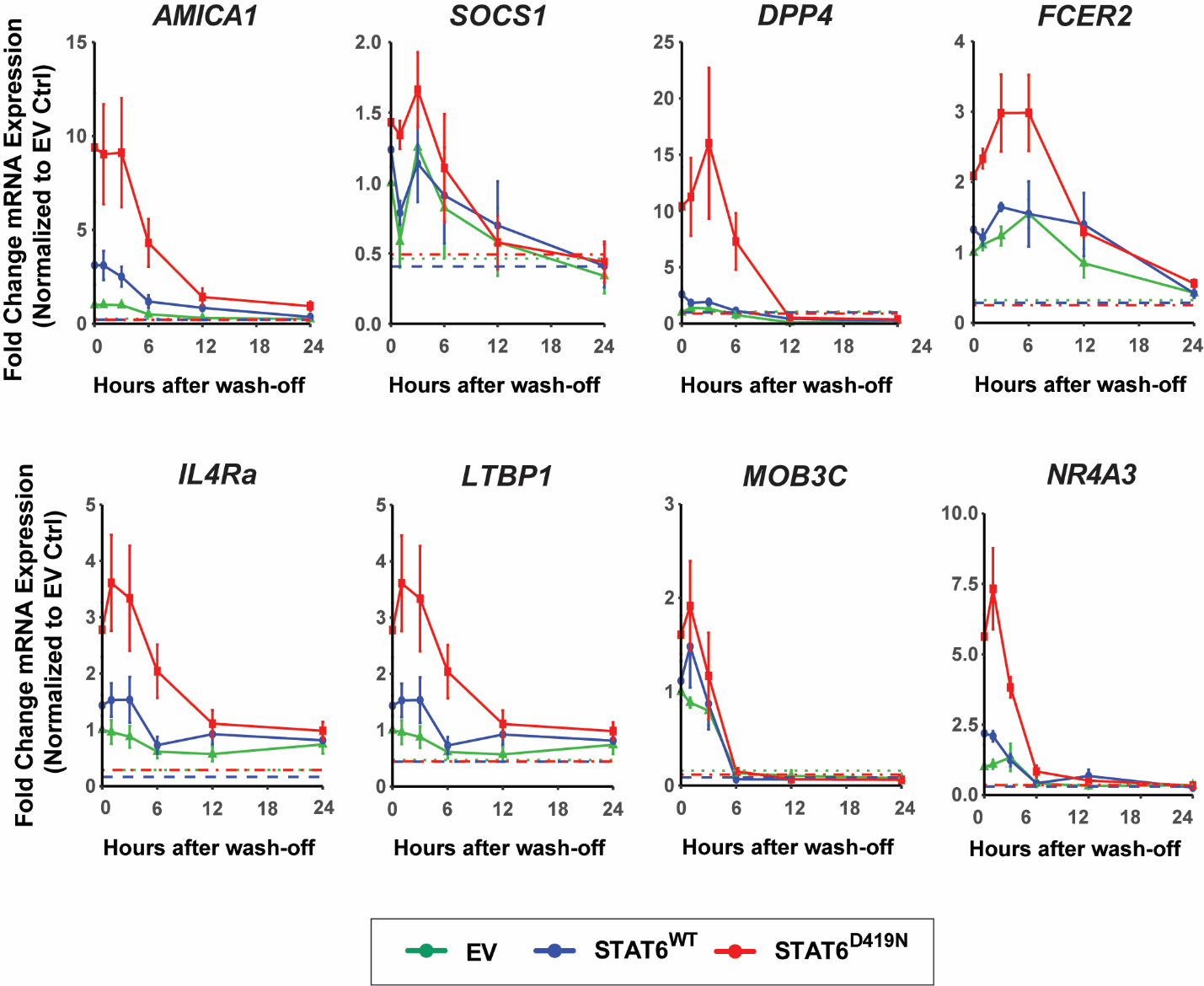
**Figure S5:**

qPCR validation of RNAseq data. Cells were stimulated with IL-4 for 72hr, then IL-4 was washed off (cells were spun down and media was replaced). RNA was extracted from OCI-Ly8-EV, OCI-Ly8-*STAT6^WT^*, and OCI-Ly8-*STAT6^D419N^* cells 0, 1, 3, 6, 12, and 24hr post-IL-4 wash off, and qPCR was performed.


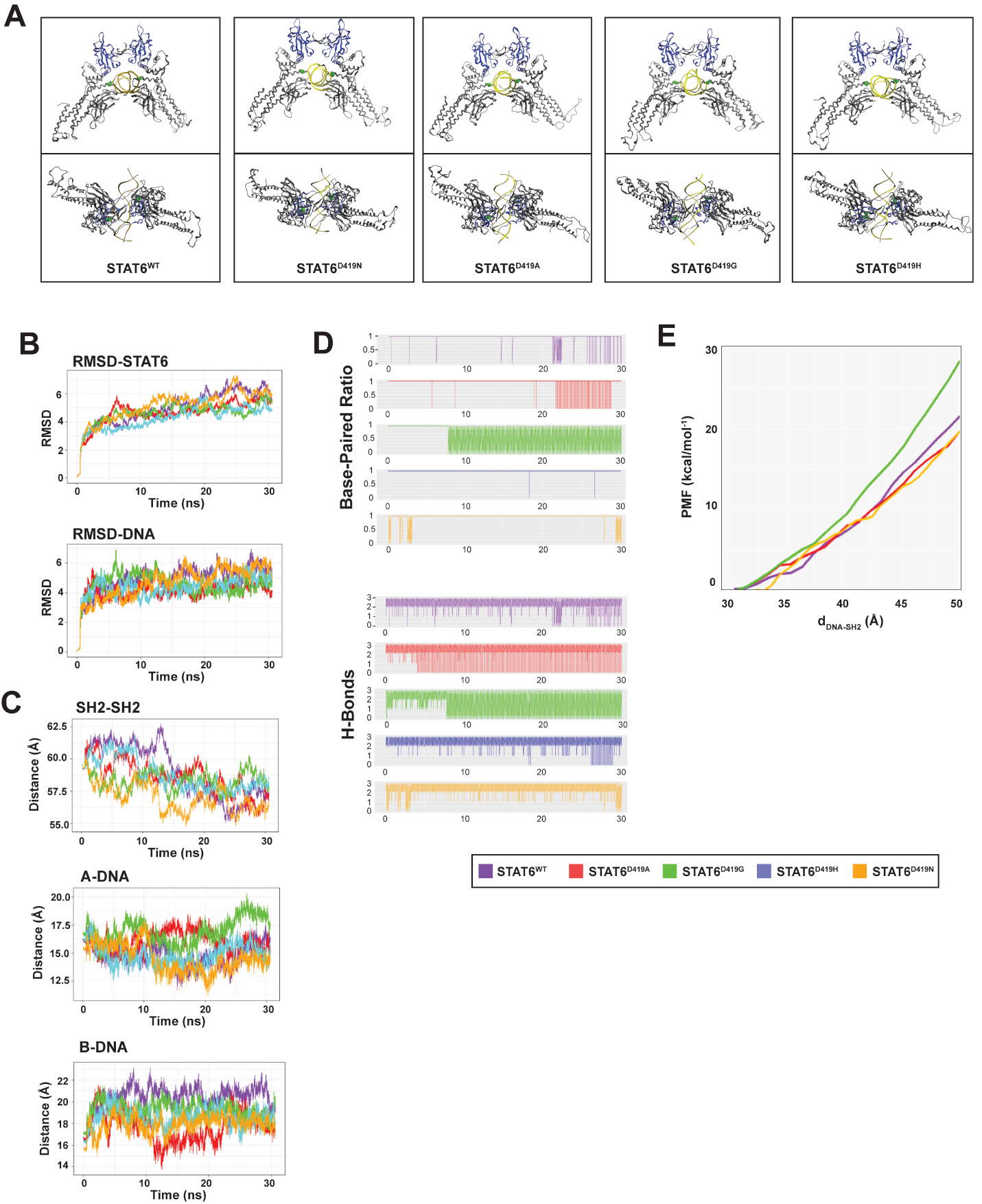


**Figure S6:**

**A.** Ribbon diagrams showing the DNA-bound conformation of STAT6^WT^ and STAT6^D419A/G/HN^ mutants. **B.** Root Mean Square Deviation (RMSD) of STAT6 and DNA backbone, monitored to guarantee to convergence to a metastable state for each STAT6-DNA complex. **C.** Molecular dynamic simulations showing STAT6 DNA binding dynamics. SH2-SH2 shows the distance between SH2 domains, measured between their centre of mass, A-DNA shows the distance between one of the D419 residues and the DNA centre of mass (chain A, resID 291), and B-DNA shows the distance between the other D419 residue and the DNA centre of mass (chain B, resID 814). **D.** Evolution of the number of DNA intermolecular hydrogen bonds and the paired bases ratio along the molecular dynamics simulation. **E.** Potential of Mean Force (PMF) obtained after umbrella sampling simulations: PMF involving the DNA unbinding process is shown. Legend applies to figures **B-E**.


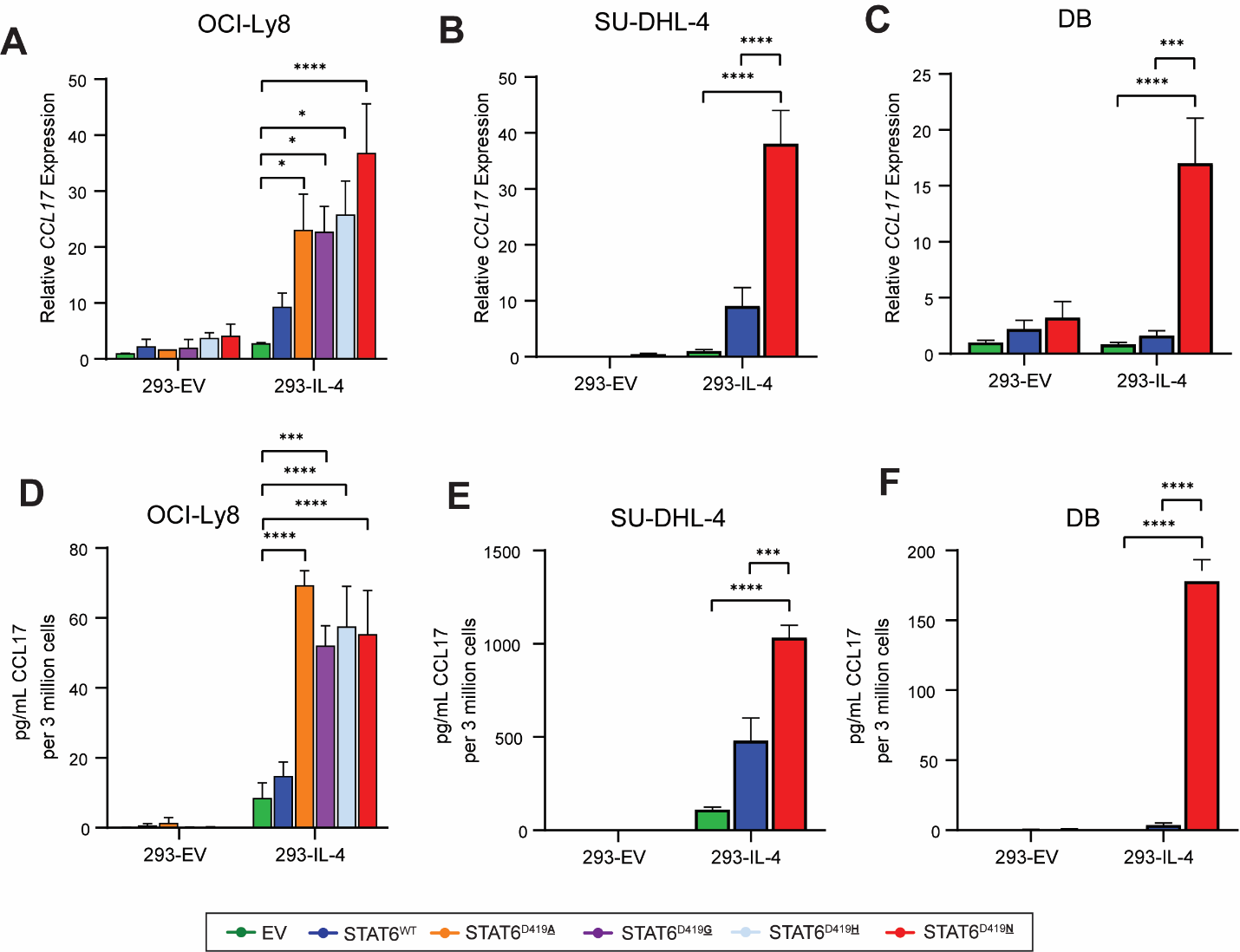
**Figure S7:**

**A.** qPCR showing *CCL17* transcription is increased in OCI-Ly8 *STAT6^D419A/G/H/N^* cells upon IL-4 stimulation. Data consist of 3 biological replicates. **B-C.** qPCR showing *CCL17* transcription is increased in SU-DHL-4 and DB *STAT6^D419N^* cells upon IL-4 stimulation. Data consists of 4 biological replicates. **D.** ELISA showing CCL17 secretion is increased in OCI-Ly8 *STAT6^D419A/G/H/N^* cells compared to STAT6^WT^ and EV control cells upon IL-4 stimulation. Data consists of 3 biological replicates in technical duplicate. **E-F**. ELISA showing CCL17 secretion is increased in SU-DHL-4 and DB *STAT6^D419N^* cells compared to STAT6^WT^ and EV control cells upon IL-4 stimulation. Data consists of 4 biological replicates in technical duplicate.

(2way ANOVA; * = *p* < 0.05, ** = *p* < 0.01, *** = *p* < 0.005, **** = *p* < 0.001).


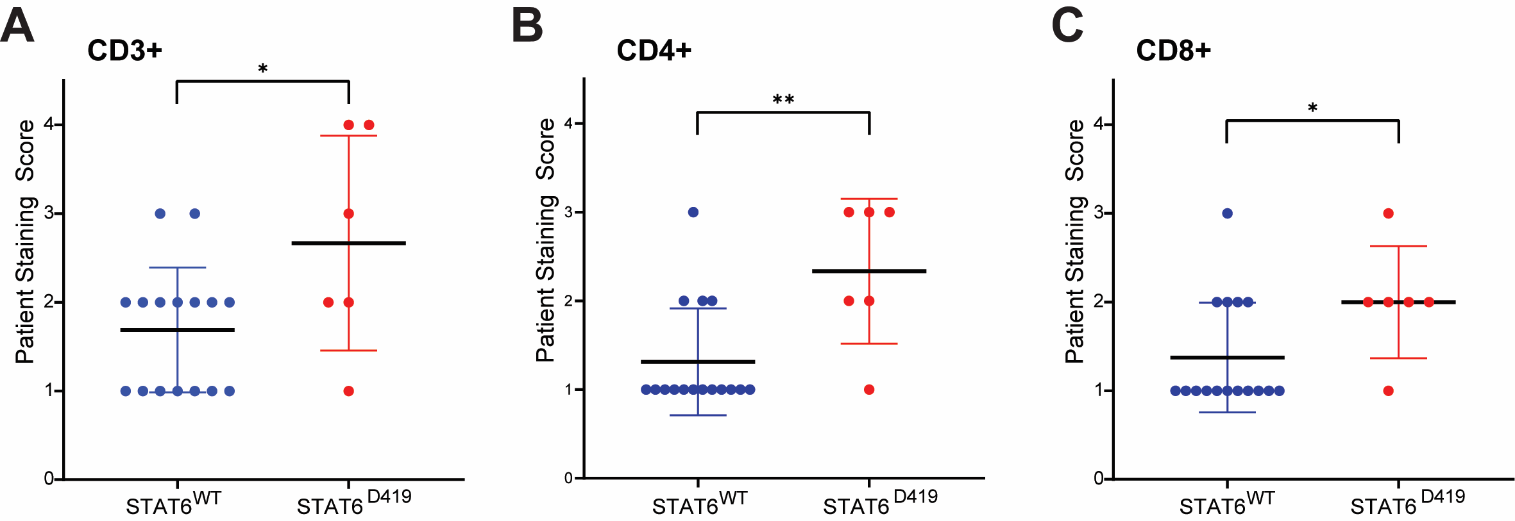


**Figure S8:**

**A-C.** CD3, CD4, or CD8 staining intensity score was compared between STAT6^WT^ and STAT6^D419^ mutant patients. Staining was scored by a blinded pathologist; 1 = negative staining, 2 = weak staining, 3 = moderate staining, 4 = strong staining. (Two-tailed unpaired t-test; * = *p* < 0.05, ** = *p* < 0.01).

**SUPPLEMENTAL REFERENCES**

1. Gu Z. Complex heatmap visualization. iMeta **2022**;1(3):e43 doi <https://doi.org/10.1002/imt2.43>.

2. Arthur SE, Jiang A, Grande BM, Alcaide M, Cojocaru R, Rushton CK*, et al.* Genome-wide discovery of somatic regulatory variants in diffuse large B-cell lymphoma. Nature Communications **2018**;9(1):4001 doi 10.1038/s41467-018-06354-3.

3. Morin RD, Mungall K, Pleasance E, Mungall AJ, Goya R, Huff RD*, et al.* Mutational and structural analysis of diffuse large B-cell lymphoma using whole-genome sequencing. Blood **2013**;122(7):1256-65 doi 10.1182/blood-2013-02-483727.

4. Dreval K, Hilton LK, Cruz M, Shaalan H, Ben-Neriah S, Boyle M*, et al.* Genetic Subdivisions of Follicular Lymphoma Defined by Distinct Coding and non-coding Mutation Patterns. Blood **2023** doi 10.1182/blood.2022018719.

5. Hübschmann D, Kleinheinz K, Wagener R, Bernhart SH, López C, Toprak UH*, et al.* Mutational mechanisms shaping the coding and noncoding genome of germinal center derived B-cell lymphomas. Leukemia **2021**;35(7):2002-16 doi 10.1038/s41375-021-01251-z.

6. Schmitz R, Wright GW, Huang DW, Johnson CA, Phelan JD, Wang JQ*, et al.* Genetics and Pathogenesis of Diffuse Large B-Cell Lymphoma. N Engl J Med **2018**;378(15):1396-407 doi 10.1056/NEJMoa1801445.

7. Thomas N, Dreval K, Gerhard DS, Hilton LK, Abramson JS, Ambinder RF*, et al.* Genetic subgroups inform on pathobiology in adult and pediatric Burkitt lymphoma. Blood **2023**;141(8):904-16 doi 10.1182/blood.2022016534.

8. Kim S, Scheffler K, Halpern AL, Bekritsky MA, Noh E, Källberg M*, et al.* Strelka2: fast and accurate calling of germline and somatic variants. Nat Methods **2018**;15(8):591-4 doi 10.1038/s41592-018-0051-x.

9. Wilm A, Aw PP, Bertrand D, Yeo GH, Ong SH, Wong CH*, et al.* LoFreq: a sequence-quality aware, ultra-sensitive variant caller for uncovering cell-population heterogeneity from high-throughput sequencing datasets. Nucleic Acids Res **2012**;40(22):11189-201 doi 10.1093/nar/gks918.

10. Benjamin D, Sato T, Cibulskis K, Getz G, Stewart C, Lichtenstein L. Calling Somatic SNVs and Indels with Mutect2. bioRxiv **2019**:861054 doi 10.1101/861054.

11. Patro R, Duggal G, Love MI, Irizarry RA, Kingsford C. Salmon provides fast and bias-aware quantification of transcript expression. Nature Methods **2017**;14(4):417-9 doi 10.1038/nmeth.4197.

12. Love MI, Huber W, Anders S. Moderated estimation of fold change and dispersion for RNA-seq data with DESeq2. Genome Biology **2014**;15(12):550 doi 10.1186/s13059-014-0550-8.

13. Morin RD, Assouline S, Alcaide M, Mohajeri A, Johnston RL, Chong L*, et al.* Genetic Landscapes of Relapsed and Refractory Diffuse Large B-Cell Lymphomas. Clin Cancer Res **2016**;22(9):2290-300 doi 10.1158/1078-0432.Ccr-15-2123.

14. Wright GW, Huang DW, Phelan JD, Coulibaly ZA, Roulland S, Young RM*, et al.* A Probabilistic Classification Tool for Genetic Subtypes of Diffuse Large B Cell Lymphoma with Therapeutic Implications. Cancer Cell **2020**;37(4):551-68.e14 doi 10.1016/j.ccell.2020.03.015.
